# Functional monocentricity with holocentric characteristics and chromosome-specific centromeres in a stick insect

**DOI:** 10.1126/sciadv.ads6459

**Published:** 2025-01-01

**Authors:** William Toubiana, Zoé Dumas, Patrick Tran Van, Darren J. Parker, Vincent Mérel, Veit Schubert, Jean-Marc Aury, Lorène Bournonville, Corinne Cruaud, Andreas Houben, Benjamin Istace, Karine Labadie, Benjamin Noel, Tanja Schwander

**Affiliations:** ^1^ Department of Ecology and Evolution, University of Lausanne, 1015 Lausanne, Switzerland.; ^2^ Institut Curie, PSL Research University, INSERM U932, Paris, France.; ^3^ School of Natural Sciences, Bangor University, Bangor, UK.; ^4^ Leibniz Institute of Plant Genetics and Crop Plant Research (IPK) Gatersleben, 06466 Seeland, Germany.; ^5^ Génomique Métabolique, Genoscope, Institut François Jacob, CEA, CNRS, Univ Evry, Université Paris-Saclay, Evry 91057, France.; ^6^ Genoscope, Institut François Jacob, CEA, CNRS, Univ Evry, Université Paris-Saclay, Evry 91057, France.; ^7^ Department of Molecular and Cellular Biology, University of Geneva, Geneva, Switzerland.

## Abstract

Centromeres are essential for chromosome segregation in eukaryotes, yet their specification is unexpectedly diverse among species and can involve major transitions such as those from localized to chromosome-wide centromeres between monocentric and holocentric species. How this diversity evolves remains elusive. We discovered within-cell variation in the recruitment of the major centromere protein CenH3, reminiscent of variation typically observed among species. While CenH3-containing nucleosomes are distributed in a monocentric fashion on autosomes and bind tandem repeat sequences specific to individual or groups of chromosomes, they show a longitudinal distribution and broad intergenic binding on the X chromosome, which partially recapitulates phenotypes known from holocentric species. Despite this variable CenH3 distribution among chromosomes, all chromosomes are functionally monocentric, marking the first instance of a monocentric species with chromosome-wide CenH3 deposition. Together, our findings illustrate a potential transitional state between mono- and holocentricity or toward CenH3-independent centromere determination and help to understand the rapid centromere sequence divergence between species.

## Teaser

The centromere defining histone variant CenH3 binds to a single spot on autosomes but longitudinally on the X chromosome.

## INTRODUCTION

Chromosome segregation is a fundamental and conserved cellular process in eukaryotes, ensuring the transmission of genetic material to daughter cells during mitotic and meiotic cell divisions (*1*). It is governed by specialized chromosomal regions known as centromeres, where a unique histone H3 variant, referred to as CenH3 (or CenpA), is recruited to nucleosomes to replace the canonical H3 histone (*2*, *3*). In turn, CenH3 facilitates the assembly of the kinetochore protein complex, which mediates the attachment of spindle microtubules (*4*–*6*). In the majority of eukaryotes, CenH3 binds to DNA sequences at a single, well-defined centromere region on each chromosome. Centromere sequences in these so-called monocentric species are typically composed of tandem or interspaced repeats, which are absent or rare in noncentromeric regions of the genome (*7*). By contrast, multiple lineages have independently evolved a holocentric chromosome structure, where CenH3 but also kinetochore proteins and microtubules attach along the entire length of all chromosomes (*8*, *9*). In these lineages, centromere sequences vary, and can comprise repeated DNA motifs akin to those in monocentric species, or more complex DNA structures forming broad intergenic or poorly transcribed domains (*10*–*14*). Irrespective of the variation that exists between species, all chromosomes within a species always share the same centromere configuration such that they either segregate in a monocentric or holocentric manner. This uniformity in centromere configuration has notably led to the argument that centromere diversity and complexity arise from discrete evolutionary transitions, without intermediate states (*15*).

While investigating the evolution of centromeres within the stick insect genus *Timema*, we uncovered a unique spatial organization and recruitment dynamic of the CenH3 protein, with phenotypes reminiscent of both mono- and holocentric species. We substantiated our intriguing cytological observations with chromatin immunoprecipitation and sequencing (ChIP-seq) data, which further revealed that CenH3 binds to notably different sequences, encompassing both tandem repeats and more complex DNA structures. Last, we also observed unexpected variability in the role of CenH3 to assemble kinetochores and recruit spindle microtubules, thus questioning its primary role in defining centromeres. Together, our findings indicate a possible intermediate state in transitions from mono- to holocentricity, or toward a CenH3-independent centromere definition.

## RESULTS

### CenH3 distribution varies between autosomes and the X chromosome

**Fig. 1.**
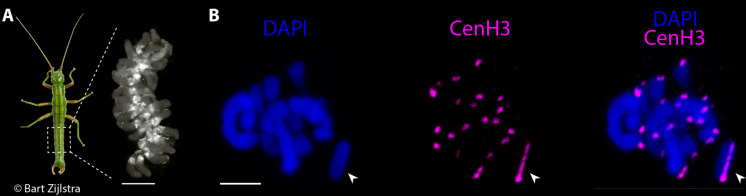
Differential CenH3 binding between autosomes and the X chromosome at metaphase I during male meiosis. (**A**) Image of a *Timema* male and a gonad containing meiotic and spermatid cells at various stages of maturation. The dashed square highlights the abdominal region housing the pair of gonads. Scale bar corresponds to 400 micrometers. Photo credit: B. ZIJLSTRA, bartzijlstra.com. (**B**) Ring and rod bivalents of paired homologous autosomes in metaphase I. The univalent sex chromosome (X chromosome) is indicated by the arrowheads. At this stage, CenH3 exhibits a monocentric distribution on the autosomes and a longitudinal distribution on the X chromosome. Scale bars, 5 μm.

*Timema* is a genus of stick insects with multiple independent transitions to asexuality (female-producing parthenogenesis) (*16*). This context provides a unique opportunity to investigate the evolution of centromeres under different selective conditions. While characterizing centromeres in different *Timema* species, we conducted immunostaining on male gonads of *T. douglasi* using a custom antibody for the CenH3 protein (Fig. 1). As CenH3 plays a central role in centromere identity and chromosome segregation in eukaryotes, our focus was directed toward cells in meiotic metaphase, when spindle microtubules bind to centromeres before segregation initiates (*4*).

**Fig. 2.**
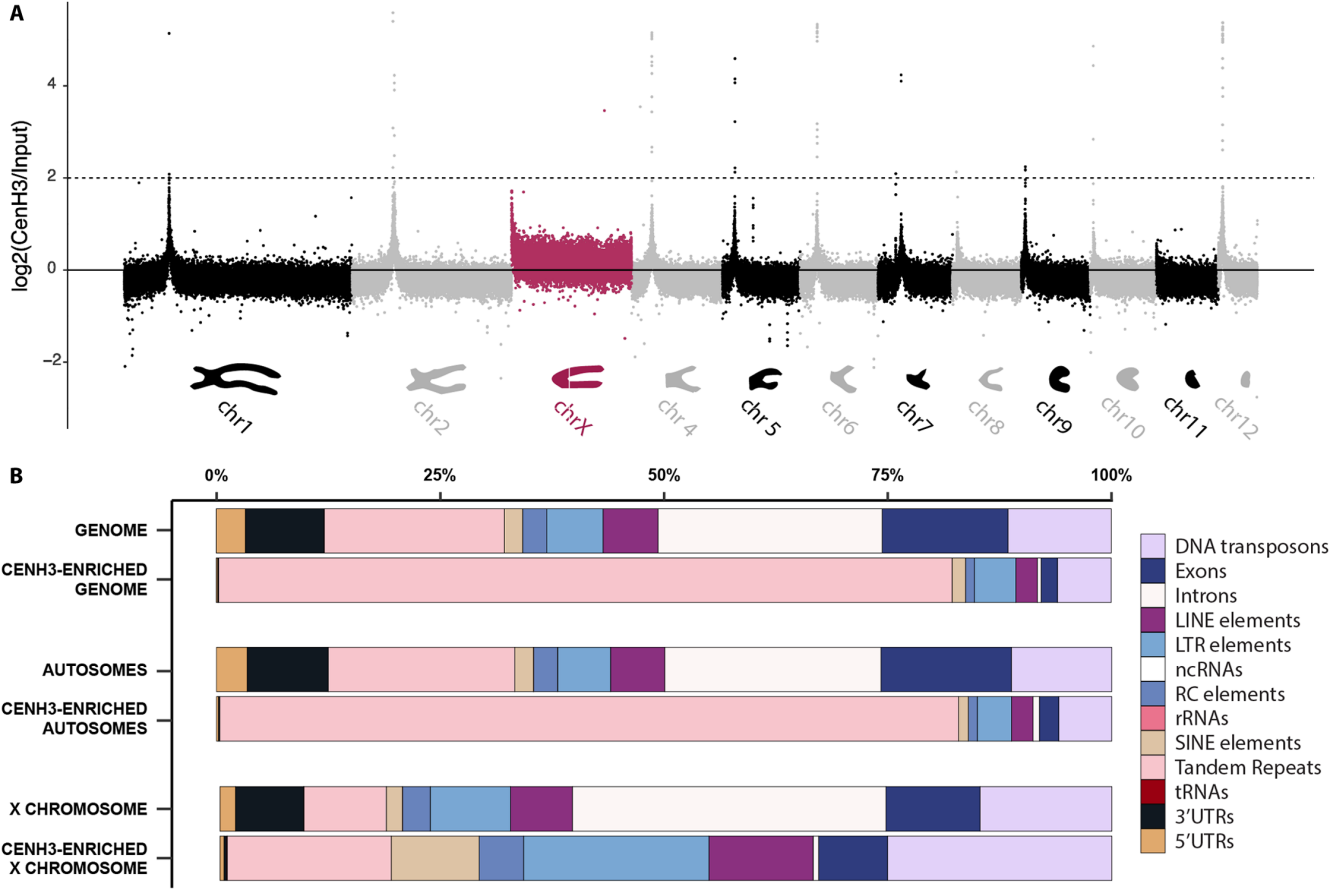
CenH3-ChIP signal corroborates differences in CenH3 distribution between autosomes and the X chromosome. (**A**) Depiction of the CenH3 directed ChIP-signal in nonoverlapping 10-kb windows log2-transformed coverage ratio of CenH3 immunoprecipitation-derived reads versus control reads without immunoprecipitation (input) along each chromosome (*x* axis). The schematic representation of the chromosome morphologies [modified from (*17*)] indicates approximate locations of the primary constrictions. The dashed line denotes the threshold used to define CenH3-enriched regions (i.e., centromere windows; see Materials and Methods). (**B**) Representation of 13 genomic features observed genome-wide, on autosomes, and the X chromosome, and enriched in CenH3-directed ChIP.

During metaphase I, we observed notable differences in CenH3 distribution between the autosomes and the X chromosome. On each of the 22 autosomes, CenH3 formed a single, distinct focus (Fig. 1B). This pattern is characteristic of species with monocentric chromosomes and was expected for *Timema* given their karyotypes with chromosomes featuring clear primary constrictions (*17*). Conversely, the univalent X chromosome exhibited binding of CenH3 as one line along its entire length (Fig. 1B), recapitulating partially the CenH3 phenotypes observed in species with holocentric chromosomes, where CenH3 binds as two lines along each chromosome (*18*, *19*). Note that *Timema* have an X0 sex chromosome system, meaning that there is a single X and no Y chromosome in males (*17*). We confirmed the different CenH3 binding patterns between the X and autosomes in a CenH3-directed ChIP-seq assay on male gonads, where we mapped ChIP and input derived sequence reads to a newly generated chromosome-level genome assembly of the same species (SAMN41832294; see Materials and Methods; fig. S1 and table S1). While each of the autosomes displayed a distinct, monocentromere-typical ChIP signal, the X chromosome was characterized by an enhanced signal distributed over its entire length, with a somewhat increased intensity at one chromosome end (Fig. 2A).

Given the highly unexpected CenH3 distribution on the X chromosome of *T. douglasi* males, we investigated whether it was specific to the focal species or a general feature in the *Timema* genus. We used males from three additional species, which cover the phylogenetic breadth of the genus, to conduct CenH3 staining as described for *T. douglasi*. Our cytological observations were consistent across all species (fig. S2), indicating that the different CenH3 distribution between autosomes and the X chromosome is a conserved feature in *Timema* males.

### The longitudinal CenH3 recruitment is dynamic and extends beyond the X chromosome in early male meiosis

**Fig. 3.**
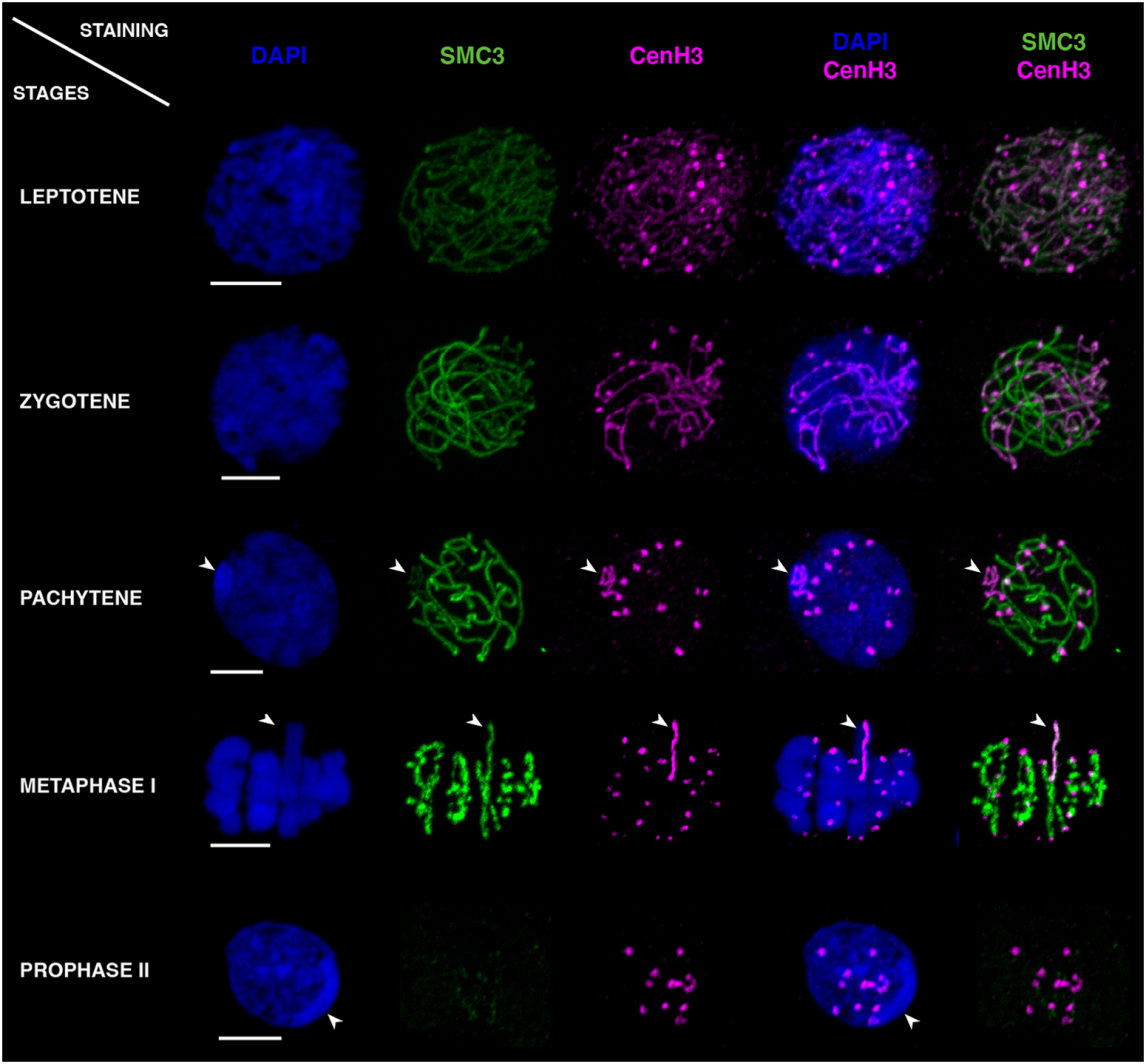
Dynamics of CenH3 and SMC3 distributions along *T. douglasi* chromosomes during prophases I and II. The labeling highlights DNA with 4′,6-diamidino-2-phenylindole (DAPI) staining in blue, SMC3 in green, and CenH3 in magenta. During the leptotene and zygotene stages, CenH3 and SMC3 colocalize along the entire length of all chromosomes. CenH3 then retracts to single foci on all chromosomes except the X during pachytene, while SMC3 remains distributed along the entire length of all chromosomes until metaphase I. Prophase II is marked by the disappearance of the longitudinal distribution of CenH3 on the X chromosome and SMC3 retracting to single foci near the monocentrically distributed CenH3. When discernible, the X chromosome is indicated by arrowheads. Scale bars, 5 μm.

To elucidate the cellular mechanism(s) governing CenH3 distribution along the X chromosome, we examined CenH3 localizations at earlier meiotic stages. We assigned individual cells to specific stages using the structural maintenance of chromosomes protein 3 (SMC3), a subunit of the cohesin complex that aids in recognizing key steps during meiosis (*20*). Unexpectedly, we found that at the onset of meiosis, the longitudinal CenH3 distribution is not restricted to the X chromosome. Instead, CenH3 is observed along the entire length of all chromosomes, colocalizing with SMC3 (Fig. 3). This longitudinal CenH3 recruitment was most prominent during the leptotene and zygotene stages of prophase I when sister chromatid cohesion was established and homologous chromosome synapsis was only initiated (Fig. 3). As synapsis progressed, CenH3 became gradually condensed into a discrete focus on each autosome (Fig. 3, zygotene and pachytene stages), while its longitudinal distribution persisted on the univalent X chromosome (Fig. 3, pachytene and metaphase I stages). Last, at the onset of meiosis II, when sister chromatid arms are no longer attached together and the cohesin complex is removed from chromosome axes, the longitudinal CenH3 distribution on the X disappeared, such that a single focus was visible on all chromosomes (Fig. 3, prophase II stage). In summary, the entry of meiosis, generally associated with cohesion of sister chromatids and chromatin compaction via loop extrusion (*21*), is characterized by CenH3 recruitment along the entire length of all chromosomes in *Timema*. Once synapsis is completed, only the X chromosome maintains a longitudinal CenH3 distribution, most likely because it does not have a homologous partner to synapse with in males. The longitudinal CenH3 phenotype mirrors the phenotype of centromere proteins during the zygotene/pachytene stages in some holocentric species. However, in the latter, centromere proteins relocalize to the outer edge of the chromosomes before metaphase I, which is not what we observe in *Timema* (*18*, *22*, *23*).

### CenH3 binding sites differ between the X and autosomes and reveal distinct autosomal centromeres

We then investigated whether the distinct CenH3 distributions on autosomes versus the sex chromosome were mirrored by distinct CenH3 binding sites. To this end, we used our CenH3-directed ChIP data on male gonads and first tested for enrichment of specific sequence categories (i.e., exons, introns, tandem repeats, DNA transposons, etc.; Fig. 2B). Enrichment patterns differed notably between the autosomes and the X. For the autosomes, the CenH3-ChIP data were predominantly composed of tandem repeat sequences, with a highly significant enrichment relative to the genomic background (Fig. 2B; χ^2^ = 1941157, degrees of freedom (DF) = 12, and *P* < 0.001; see Material and Methods). For the X chromosome, no specific sequence category was overrepresented (Fig. 2B). Instead, the CenH3-ChIP data had a reduced representation in genic regions [i.e., introns, exons, and untranslated regions (UTRs)], which largely drove the significant difference in sequence categories relative to the genomic background (Fig. 2B; χ = 189028, DF = 12, and *P* < 0.001).

**Fig. 4.**
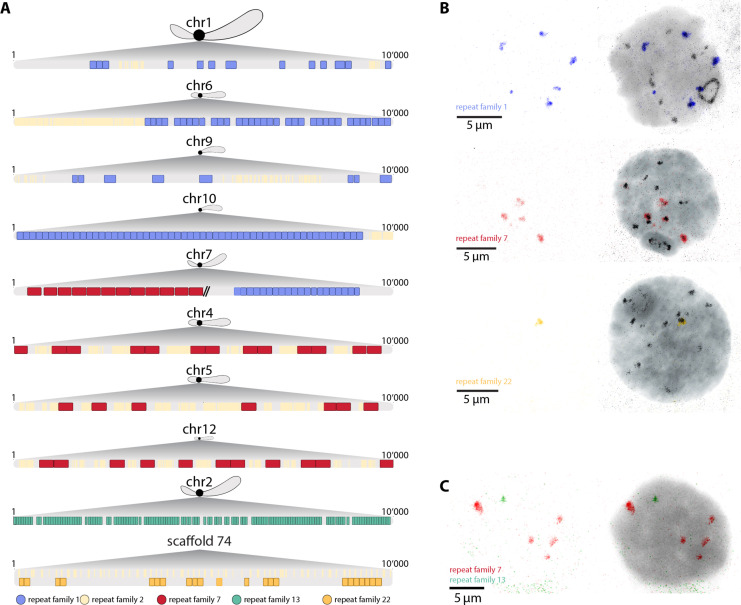
The centromere sequence composition differs between *T. douglasi* autosomes. (**A**) 10-kb regions with the most enriched CenH3 ChIP signal, color-coded with the five most abundant centromere repeat families representing over 90% of the total tandem repeat content. Chromosome drawings indicate the centromere localization within each autosome, including sub-metacentric (chromosomes 1, 2, and 7), acrocentric (chromosomes 4, 5, 6, and 12), and telocentric (or near telomeres; chromosomes 9 and 10) chromosomes. Note that the telocentric chromosomes 8 and 11 are missing because of incompletely assembled centromeres (see text). (**B**) Immuno-FISH on meiotic cells labeled with CenH3 proteins (black) and DNA probes targeting different centromere repeat families. (**C**) FISH staining highlighting the location of two centromere repeat families, revealing that they are on distinct chromosomes (nonoverlapping).

We further used our CenH3-ChIP data to characterize centromere sequences in *T. douglasi.* Given the enrichment in tandem repeats in the CenH3-ChIP data, we identified specific tandem repeat families constituting centromeres by using an assembly-based and assembly-free (i.e., k-mer based) approach (see Materials and Methods; fig. S3). For both approaches, individual repeats were grouped into families using network clustering analysis which resulted in 28 and 16 centromere repeat families, respectively, with closely matching motif sequences (fig. S3, A and C, and tables S2 and S3). Among these, six dominant repeat families (repeat families 1, 2, 7, 10, 13, and 22; table S2) collectively constituted more than 90% of the tandem repeats found in centromeric regions. One of these abundant families (repeat family 2) comprised the AACCT motif, a telomere repeat in various insect lineages [Fig. 4A, figs. S3 and S4, and table S2; (*24*, *25*)]. Four other families (repeat families 1, 7, 13, and 22) consisted of motifs ranging from 63 to 384 base pairs in size. Last, the sixth family (repeat family 10), with a motif size of ~1400 bp, was a composite of families 2 and 7 (figs. S3 and S4 and table S2) and was therefore not considered further.

We examined the representation of the five distinct repeat families (repeat families 1, 2, 7, 13, and 22) in 9 of the 11 autosomal centromeres. The incomplete assembly of centromeres of the two remaining autosomes (chromosomes 8 and 11), both telocentric (Fig. 2A), precluded their in-depth examination. The distribution of the five repeat families among the nine more complete autosomes revealed substantial differences in centromere composition (fig. S4, A and B, and table S4). In four of these nine autosomes (chromosomes 1, 6, 9, and 10), centromeres consisted of two repeat families (families 1 and 2), which were each tandemly repeated (Fig. 4A). The centromeres of three additional autosomes (chromosomes 4, 5, and 12) consisted of two repeat families (families 2 and 7) organized into higher-order repeats (Fig. 4A). One autosome (chromosome 7), contained repeats present in the centromeres of two otherwise distinct groups of autosomes (Fig. 4A), while chromosome 2 had a unique, chromosome-specific centromere sequence (Fig. 4A). Overall, repeat family 2 (corresponding to the telomeric AACCT motif) was the most broadly shared, as it was found in high abundance in the centromeres of seven autosomes (chromosomes 1, 4, 5, 6, 9, 10, and 12).

We corroborated the inferred centromere sequences for individual or groups of autosomes through a combination of fluorescence in situ hybridization (FISH) and immuno-FISH experiments. Thus, repeat family 7, which was predicted to be present in the centromeres of four autosomes (chromosomes 4, 5, 7, and 12), labeled four centromeres along with CenH3 (Fig. 4B). Repeat family 13, which was predicted to be specific to chromosome 2, labeled a single chromosome (Fig. 4C). Repeat family 22, which was not represented in the centromeres of the nine more complete autosomes but only on four unanchored scaffolds (Fig. 4A and fig. S4, A and B), also labeled a single chromosome (Fig. 4B and fig. S5). Last, repeat family 1 was predicted to be present on five autosomes and indeed labeled five autosomes and one extremity of the X chromosome (Fig. 4, A and B). Each of the examined repeat families exhibited distinct nuclear locations, indicating that they are in centromeres of different chromosomes (Fig. 4C and fig. S5). Collectively, these findings highlight the divergence of centromere sequences among different autosomes of *T. douglasi*, which mirrors the well documented rapid divergence of centromere sequences between species (*7*, *15*, *26*). The reasons why some chromosomes have unique (chromosome-specific) centromere sequences while others form groups of similar centromere sequences remain to be investigated. Admixture between divergent populations or species could result in centromere heterogeneity, yet introgression analyses in *Timema* do not point to hybrid origins of *T. douglasi* (*16*). Another possible situation favoring centromere sequence convergence among specific sets of chromosomes is nuclear compartmentalization, where certain chromosomes or certain centromeres cluster into specific regions of the nucleus (*27*, *28*). Such spatial clustering could facilitate convergent evolution between interacting centromere repeats on different chromosomes.

Regarding the X chromosome, our immuno-FISH experiments indicated that in addition to the longitudinal CenH3 distribution, one chromosome end likely harbors a localized centromere, composed of family 1 repeats (Fig. 4B). This region was also marked by an enriched CenH3 immunolabeling signal (Figs. 1B and 4B), and exhibited a slightly elevated ChIP signal at the left end of the X assembly (Fig. 2A), consistent with the telocentric location of the primary constriction.

As centromere specification can vary between mitotic and meiotic cells and be associated with different chromosomal locations of the CenH3 protein (*29*, *30*), we investigated whether the distinct CenH3 distributions between the X and autosomes were specific to meiotic cells or also present in mitotic cells. Immunolabeling of the CenH3 protein in meta- and anaphase-stage mitotic cells revealed a monocentric distribution across all chromosomes (fig. S6). We corroborated this monocentric distribution using CenH3-directed ChIP-seq based on composite somatic tissues (fig. S6). Collectively, our data indicate that the longitudinal CenH3 distribution on the X is exclusive to male meiotic cells, whereas in mitotic cells, CenH3 is recruited in a monocentric fashion at a single regional end of the X chromosome.

### Kinetochore protein distributions and microtubule attachments suggest a functionally monocentric X chromosome

**Fig. 5.**
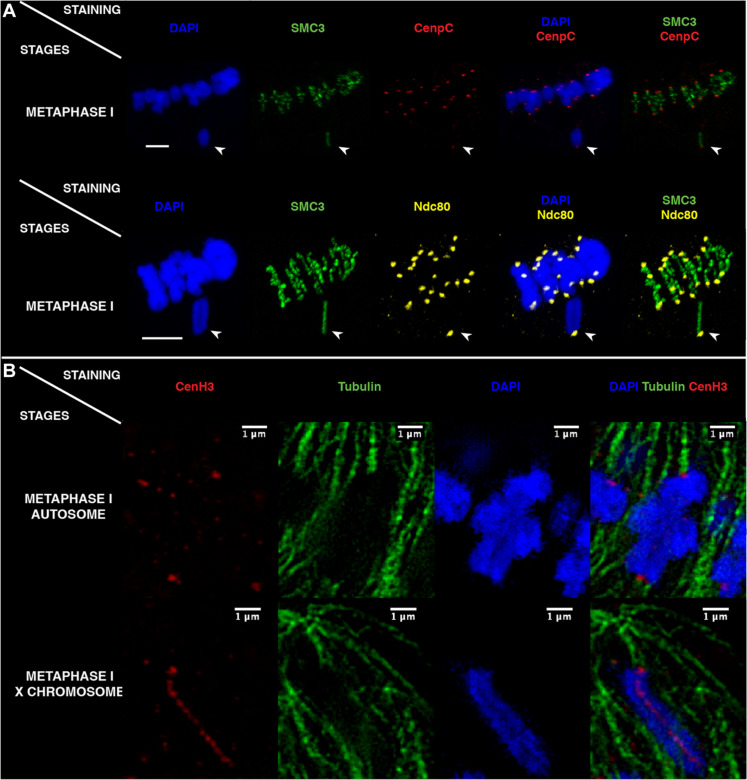
Kinetochore and spindle microtubule distributions on *T. douglasi* metaphase I chromosomes. (**A**) All chromosomes, including the X chromosome (arrowheads), showed monocentric-typical CenpC (red) and Ndc80 (yellow) signals at the poleward chromosome termini. Scale bars, 5 μm. (**B**) 3D-SIM (single slice of an image stack) visualized the monocentric microtubule attachment sites along with CenH3 on autosomes and the X chromosome during metaphase I.

To determine whether the longitudinal CenH3 distribution translates into a functionally holocentric X chromosome during segregation, we investigated the distribution of kinetochore proteins and used super-resolution three-dimensional structured illumination microscopy (3D-SIM) to localize microtubule attachment sites. As expected for a functionally monocentric X chromosome, CenpC and Ndc80 proteins (members of the inner and outer kinetochore region, respectively) were distinctly visible at a unique position on the X at metaphase I, resembling the CenH3-monocentric autosomes (Fig. 5A). Similarly, α-tubulin and CenH3 costaining analyzed using 3D-SIM [(*31*); see Materials and Methods] revealed monocentric spindle attachments to a delimited and terminal region of the X chromosome as well as autosomes (Fig. 5B). Together, these findings indicate that despite the longitudinal distribution of CenH3, the X chromosome behaves as a functionally monocentric chromosome during the first meiotic division in *Timema* males. In this context, it is possible that the disparate distributions of CenH3 and kinetochore proteins may stem from an undescribed function of the CenH3 protein such as in the regulation of univalent chromosomes, where it could help the monopolar orientation of the X chromosome or prevent the separation of the two chromatids during anaphase I (*32*, *33*).

To our knowledge, this study represents the first description of a lack of co-occurrence of CenH3 with certain kinetochore and spindle microtubule proteins, despite their presence in the same cellular environment, in both animals and monocentric species more generally. In addition to suggesting two distinct recruitment steps for centromere proteins, our observations contrast the findings in human cell lines and *Drosophila* mutants where the mis-incorporation of CenH3 leads to an ectopic assembly of partial or full kinetochore complexes (*34*, *35*).

## DISCUSSION

Centromeres and variation in centromere organization have been known since early work on karyotype evolution in plants and animals (*9*), yet our understanding of how these key structures evolve and diversify remains limited (*15*). For example, how transitions from monocentric to holocentric centromere configurations can occur over the course of evolution, and whether these transitions are sudden or gradual, is unknown (*15*). In *Timema*, the centromere histone variant CenH3 can show a longitudinal or monocentric distribution within the same cell, with the distribution varying between chromosome types and meiotic stages. This plasticity in CenH3 binding highlights the potential for rapid evolution between different centromere configurations such that it may help to understand how holocentric transitions have repetitively occurred across eukaryotes (*9*).

Specifically, our study illustrates that transitions to holocentricity can evolve gradually. For example, CenH3 could acquire the ability to bind along chromosome lengths before entailing functional holocentricity. Our findings reveal that the longitudinal distribution of CenH3 can be decoupled from the longitudinal recruitment of kinetochore proteins and holocentric segregation of the X chromosome. The disparate distributions of CenH3 and kinetochore proteins may stem from the distribution of the CenH3 proteins positioned in between the two chromatids, reminiscent of the cohesin distribution, which potentially impedes the assembly of a functional kinetochore and subsequent holocentric segregation. Holocentric lineages in plants and animals typically have their CenH3-enriched chromatin facing outward of the two chromatids such that two linear lines form on every chromosome (*8**,* *10**,* *14**,* *19**,* *36*). However, recent studies in two holocentric moth species (with CenH3-independent kinetochores) and in the holocentric plant *Luzula elegans* have also reported cohesin-like localizations for kinetochore proteins or CenH3, respectively, during early meiosis I (*18*, *22*, *23*). At metaphase I, these proteins are then relocalized to the outer edge of the chromosomes. In addition to strengthening the idea that the CenH3 phenotype in *Timema* can illustrate a possible transitional state between mono- and holocentricity, these data indicate that a transition toward holocentricity in insects may have involved the relocalization of CenH3 or other kinetochore proteins to the outer edge of the chromosomes during meiosis as a key step.

Alternatively, the disparate distributions between CenH3 and kinetochore proteins in *Timema* may represent a first step toward a CenH3-independent assembly of the kinetochore complex. CenH3-independent centromeres have repeatedly evolved within insect phylogeny, in association with transitions to holocentricity (*37**,* *38*). Under this scenario, *Timema* would represent an intermediate state between CenH3-independent holocentric lineages and CenH3-dependent monocentric lineages. More generally, the fact that CenH3 alone is not sufficient to drive kinetochore assembly and microtubule recruitment in *Timema*, as well as in some holocentric insect and plant lineages (*38**,* *39*), questions the view that CenH3 is the key epigenetic “mark” defining centromeres.

Another knowledge gap in centromere evolution is to understand how centromere sequences can evolve so rapidly among species as this should require parallel rapid evolution of DNA-centromere protein affinities (*15*, *26*). Our study contributes to filling this knowledge gap by first revealing that CenH3 binds to divergent repetitive centromere sequences on different autosomes. Similar patterns have been documented in some groups of organisms such as in *Drosophila* or the plant tribe Fabeae, where CenH3 binds to very diverse repeat sequences within a species, while in other organisms such as chicken or potato, CenH3 binds to chromosomes with repeatless and repeat-based centromeres (*40*–*43*). We also reveal that CenH3 in *Timema* can bind to broad categories of intergenic regions on the X chromosome, mirroring the centromere domains of several holocentric lineages (*12*–*14*). Such versatility in binding affinity within the same cell could facilitate a rapid turnover of centromere sequences between species, including those associated with transitions to holocentricity.

Overall, *Timema* offers a unique perspective on the evolution of centromeres, which calls for an increased phylogenetic diversity in model organisms used to study key cellular features. The fact that chromosomes generally adopt either a monocentric or holocentric configuration within a lineage has been associated with the idea that centromere complexity evolves through discrete transitions (*15*). Our cellular and molecular observations suggest that gradual transitions may be possible and involve intermediate states that can remain stable over long evolutionary timescales. Last, the turnover in centromere sequence is hypothesized to result from centromere competition during asymmetric female meiosis (*26*). Because we identify notable centromere sequence divergence between nonhomologous chromosomes that do not compete during meiosis, our findings raise the question of other drivers of sequence divergence and whether cellular processes such as chromosome interactions also contribute to centromere sequence diversity.

## MATERIALS AND METHODS

### Sample collection and reference genome generation

We used wild-collected individuals of the species *T. douglasi* (individuals for CenH3 related questions: 38°57′24.9″N 123°32′10.0″W; and individuals for the reference genome: 38°58′56.4″N 123°28′11.9″W), *T. knulli* (35°50′10.3″N 121°23′29.3″W), *T. californicum* (37°20′35.4″N 121°38′11.3″W), and *T. bartmani* (34°09′48.1″N 116°51′43.5″W). To generate the *T. douglasi* reference genome, we assembled contigs based on Nanopore and Illumina libraries (sequenced at 62× and 63× coverages, respectively) generated from a single female, and then scaffolded contigs using a Hi-C library (sequenced at 95× coverage) based on a different female. We annotated this assembly using transcriptome data from different tissues and development stages of males and females of *T. douglasi* and from a closely related species (*T. poppense*; see the “Gene annotation” section).

### Sequencing libraries

To extract high–molecular weight (HMW) DNA, we flash-froze a single female (without gut) in liquid nitrogen and ground it using a Cryomill (Retsch). We then extracted HMW DNA using a G/20 Genomic Tips kit (Qiagen) following manufacturer’s protocols. We checked DNA integrity on a pulse field agarose gel.

A total of four ONT libraries were prepared following Oxford Nanopore instructions. One library was prepared using the SQK-LSK108 ligation sequencing kit and was loaded on a MinION R9.4.1 Flow Cell, and three libraries were prepared using the SQK-LSK109 ligation sequencing kit and were loaded on PromethION R9.4.1 Flow Cells. Flow cell loading was performed according to the Oxford Nanopore protocol and resulted in 62× coverage.

A polymerase chain reaction (PCR)–free Illumina library was prepared using the Kapa Hyper Prep Kit (Roche, Basel, Switzerland), following the manufacturer’s instructions. Library was quantified by qPCR using the KAPA Library Quantification Kit for Illumina Libraries (Roche), and library profile was assessed using a High Sensitivity DNA kit on an Agilent Bioanalyzer (Agilent Technologies, Santa Clara, CA, USA). The library was then sequenced to approximately 66× coverage on an Illumina HiSeq 4000 instrument (Illumina, San Diego, CA, USA), using 150 base-length read chemistry in a paired-end mode.

Hi-C library construction using the Proximo Hi-C Kit and sequencing (250 Mio read pairs) was outsourced to Phase Genomics (Seattle). We generated ground tissue for cross-linking following the manufacturer’s protocol, using a different female than the one used for Nanopore and NovaSeq sequencing, from the same natural population.

### Assembly pipeline and parameters

Raw Oxford Nanopore reads were filtered using Filtlong v0.2.0 (https://github.com/rrwick/Filtlong) with the parameters --min_length 1000 --keep_percent 90 --target_bases 69050000000. The filtered Nanopore reads were then assembled into contigs using Flye version 2.8.1 (*44*) with --genome-size 1.3 Gbp. All Nanopore reads were mapped against the contigs using minimap2 version 2.19 (*45*) with the parameters -c -x map-ont and a first step of polishing was performed using Racon version 1.4.3 (*46*). Three additional rounds of polishing were then conducted using the Illumina short reads. The short reads were aligned to the contigs using BWA mem version 0.7.17 (*47*) and polishing was performed using Pilon version 1.23 (*48*).

The assembly was decontaminated using BlobTools version 1.0 (*49*) under the taxrule “bestsumorder.” Hit files were generated after a blastn version 2.10.1+ against the National Center for Biotechnology Information (NCBI) nt database, searching for hits with an e-value below 1 × 10^−25^ (parameters: -max_target_seqs 10 -max_hsps 1 -evalue 1 × 10^−25^). Contigs without hits to metazoans were removed. Haplotypic duplications were filtered out: filtered reads were mapped against the decontaminated genome using minimap2 and haplotigs were detected with Purge Haplotigs version 1.1.1 (*50*) using the parameters -l 3 -m 17 -h 190 -j 101 following the recommendations by (*50*).

For scaffolding, Hi-C reads were mapped to the haploid genome using Juicer version 1.6 (*51*) with the restriction site Sau3AI. Chromosome-level scaffolding was then performed using 3D-DNA version 180922 (*52*) with the parameters --editor-coarse-resolution 25000, as recommended by the authors. The resulting Hi-C contact matrices were visualized with Juicebox and polished following the recommendations by (*51*). The completeness of the assembly was assessed with BUSCO version 5.1.2 (*53*) and the insecta_odb10 dataset using the --long and --augustus parameters.

To identify the X chromosome in our assembly, we used a coverage approach. We compared coverage between males and females because *Timema* have XX/X0 sex determination (*17*) and males are expected to show half of the female coverage at the X chromosome. We mapped five female [SRS7637469, SRS7637489, SRS7637497, SRS7638280, and SRS7638278 from (*54*)] and two male samples [BioProject PRJNA808673 from (*55*)] to our scaffolded genome, which allowed us to unambiguously identify the third largest scaffold as the X chromosome (fig. S1).

### Genome annotation

#### Gene annotation

The *T. douglasi* genome was annotated using a combination of ab initio gene prediction, protein homology, and RNA sequencing (RNAseq) using the Braker2 pipeline version 2.1.6 (*56*). To begin, the genome assembly was soft-masked using RepeatModeler (version 2.0.2, options: -LTRStruct, -engine ncbi) and RepeatMasker (version 4.1.2, options: -engine ncbi, -xsmall). For protein evidence, we used the arthropod protein sequences from OrthoDB version 10.1 (*57*) and the predicted protein sequences for *Timema* from our previous genome assemblies (*54*). For RNAseq evidence, we used publicly available RNAseq data from *T. douglasi* and *T. poppense* (BioProject accessions: PRJNA380865, PRJNA1128519, and PRJNA1128554). This is a total of 376 RNAseq libraries (364 paired-end and 12 single-end) covering 117 different life stages, tissue, and sex combinations. Reads were quality trimmed with Trimmomatic (version 0.39, options: ILLUMINACLIP:3:25:6 LEADING:9 TRAILING:9 SLIDINGWINDOW:4:15 MINLEN:80) (*58*) before mapping to the genome assembly with STAR (version 2.7.8a, options: --twopassMode Basic) (*59*). Braker2 was run using protein evidence and RNAseq separately with the gene predictors Augustus version 3.4.0 (*60*) and Genemark version 4.72 (*61*). Following the RNAseq run, UTR predictions were added to the RNAseq gene predictions using GUSHR version 1.0 (*62*) in Braker2 (--addUTR=on). The separate gene predictions were then merged using TSEBRA version 1.0.3 (*63*) using the pref_braker1.cfg configuration file, which weights RNAseq evidence more strongly than the default option. We then ran BUSCO (version 5.3.2, insecta_odb10) on the gene regions annotated by Braker2 and on the whole genome assembly. Any genes found by BUSCO but missed by Braker were then added to the annotation (48 genes). ncRNA genes were predicted using Infernal (version 1.1.2, minimum e-value 1e-10) (*64*). Gene ontology terms for protein-coding gene predictions were obtained using blastP within OmicsBox version 3.1.2, default parameters) to blast the nr Drosophila melanogaster database (taxonomy filter: 7227).

#### Transposable element and tandem repeat annotations

Transposable elements were annotated using RepeatModeler2 version 2.0.3 (*65*). A library of consensus sequences of repeats was built and annotated using 11 *Timema* assemblies. Structural detection of LTR elements was activated using the -LTRStruct option. To reduce redundancy, all consensus sequences were clustered using a 80% identity threshold (mmseqs22 v13; -k 0 --cov-mode 1) (*66*). For each resulting cluster, only the longest sequence was then kept in the library. To retrieve repeat positions in the genome assembly, consensus sequences from the nonredundant library were mapped to the genome using RepeatMasker3 (version 4.1.2, -no_is) (*67*).

Tandem repeat sequences were annotated with Tandem Repeat Finder (TRF) version 4.09.1 (*68*), using the parameters matching weight = 2, mismatch penalty = 7, indel score = 7, match probability = 80, indel probability = 10, minimum alignment score = 50, and motif size up to 2000 bp.

### Immunostaining

Male *Timema* gonads exhibit a grape-like appearance, with the shoot containing mature sperm cells and the grape forming an oval structure encompassing cells in various meiotic stages (Fig. 1A). Adult gonads were dissected in 1× phosphate-buffered saline (PBS), fixed in a solution consisting of 2% paraformaldehyde and 0.1% Triton X-100 for 15 min, and then 4 to 5 “grapes” were gently squashed onto poly-l-lysine–coated slides before being rapidly immersed in liquid nitrogen. After a 20-min incubation in PBS, slides were subjected to blocking with 3% bovine serum albumin (BSA) blocking buffer for a minimum of 30 min. For immunostaining, slides were incubated with diluted primary antibodies (except for the already fluorescently labeled SMC3 antibody used in the following step) overnight at 4°C within a humid chamber. Detailed information for all antibodies used are provided in table S5. Subsequently, slides underwent three 5-min washes in PBS, followed by a 1-hour incubation with the secondary antibody, diluted at a ratio of 1:200 in 3% BSA, at room temperature (RT). Slides were then washed thrice for 5 min, followed by a 10-min wash in 1× PBS. A blocking step was performed using diluted normal rabbit serum (NRS 5%) for 30 min at RT, followed by a 10-min wash in 1× PBS. Subsequently, slides were incubated for 1-hour at RT with the SMC3 antibody diluted at 1:100. Last, slides were washed three times for 5 min in PBS and mounted in 4′,6-diamidino-2-phenylindole (DAPI)/Vectashield (50:50) media.

For the immuno-FISH protocol, we introduced a postfixation step, involving incubation in a solution of 2% paraformaldehyde and 0.1% Triton X-100 for 15 min, before proceeding with the FISH protocol as detailed below (without the fixation and freezing steps).

#### Fluorescent in situ hybridization

Tissues were fixed and squashed on slides as described for the immunostaining protocol. After a rapid freezing step in liquid nitrogen, coverslips were removed and slides immersed in PBS containing 0.1% Tween 20 for 20 min. For the hybridization step, 1 μl of each probe (at a concentration of 100 ng/μl) was diluted in 20 μl of 1.1× hybridization buffer (composed of 10 μl of formamide, 4 μl of 50% dextran sulfate, 2 μl of 20× saline-sodium citrate buffer (SSC), and 4 μl of ultrapure water). This probe/hybridization buffer mixture was added to the slide and covered with a coverslip. The slides with coverslips were then heat-shocked for 1 min at 95°C and incubated at 30°C overnight within a humid chamber. Last, slides were subjected to three 5-min washes in 4× SSCT (200 ml of 20× SSC, 0.1% Triton X-100, 799 ml of ultrapure water) and three additional 5-min washes in 0.1× SSC [5 ml of 20× SSC and 995 ml of ultrapure water] before mounting in DAPI/Vectashield (50:50) media.

### Image acquisition

All acquisitions characterizing CenH3 and kinetochore phenotypes during male meiosis were performed using the Zeiss LSM 880 airyscan confocal microscope equipped with a 60×/oil immersion objective. All acquisitions were produced by the superimposition of focal planes. Postprocessing, including croppin and pseudocoloring, was carried out using Fiji (*69*).

To detect the ultrastructural organization of chromosomes, CenH3, and tubulin signals and to pinpoint spindle microtubule attachment points at a resolution of ~120 nm (super-resolution achieved with a 488-nm laser excitation), spatial SIM (3D-SIM) was performed with a 63×/1.4 Oil Plan-Apochromat objective of an Elyra 7 microscope system and the software ZENBlack (Carl Zeiss GmbH). Images were captured separately for each fluorochrome using the 561-, 488-, and 405-nm laser lines for excitation and appropriate emission filters (*70**,* *71*). All acquisitions were produced by the superimposition of focal planes and postprocessing, including editing, cropping, and pseudocoloring, was carried out using Fiji (*69*).

### Chromatin preparation

To identify CenH3 binding sequences during *Timema* male meiosis, we first performed a chromatin preparation on dissected testes immediately frozen in liquid nitrogen. Eighty-five milligrams of frozen tissues were transferred to a 2-ml Eppendorf tube, homogenized by cryogenic grinding (CryoMill; Retsch GmbH) using a specific regimen (2× 60 s, 25 Hz, resting 30 s, and 5 Hz) and sequentially diluted five times with 1 ml of cross-linking solution composed of 50 mM Hepes (pH 7.9), 1 mM EDTA (pH 8), 0.5 mM EGTA (pH 8), 100 mM NaCl, and 1% formaldehyde. The 1-ml solutions were successively transferred to a 15-ml Falcon tube and subjected to rotation at RT for 12 min. The cross-linking reaction was stopped by pelleting nuclei for 2 min at 2000*g*, followed by replacement of cross-linking solution with a stop solution and rotation for 10 min. The stop solution contained 1× PBS, 125 mM glycine, and 0.01% Triton X-100. Nuclei were then subjected to washing steps in solution A [10 mM Hepes (pH 7.9), 10 mM EDTA (pH 8), 0.5 mM EGTA (pH 8), and 0.25% Triton X-100] and solution B [10 mM Hepes (pH 7.9), 1 mM EDTA (pH 8), 0.5 mM EGTA (pH 8), 0.01% Triton X-100, and 200 mM NaCl] during 10 min each at RT. Each washing step was followed by 2-min centrifugation at 2000*g* upon which the supernatant was discarded. After the second centrifugation, nuclei were suspended in 100 μl of radioimmunoprecipitation assay (RIPA) buffer [10 mM tris-HCl (pH 8), 140 mM NaCl, 1 mM EDTA (pH 8), 1% Triton X-100, 0.1% SDS, 0.1% sodium deoxycholate, and 1× cOmplete protease inhibitor cocktail] and transferred to adaptive focus acoustics (AFA) microtubes for sonication. Sonication was performed in a Covaris S220 sonicator for 5 min with a peak incident power of 140 W, a duty cycle of 5%, and 200 cycles per burst. The sonicated chromatin was transferred to a 1.5-ml Eppendorf tube and centrifuged at maximal speed for 10 min at 4°C, before aliquoting the supernatant to 10-μl input and 90-μl ChIP samples.

### Chromatin immunoprecipitation and sequencing

ChIP was carried out using 5 μl of CenH3 antibody against 45 μl of the ChIP sample filled up to 1 ml with RIPA solution and incubated overnight at 4°C. The next day, Protein A Dynabeads (25 μl; Thermo Fisher Scientific, 100-01D and 100-03D) were added for 3 hours at 4°C, and subsequently washed eight times during 10 min: once with RIPA, four times with RIPA with 500 mM NaCl, once in LiCl buffer [10 mM tris-HCl (pH 8), 250 mM LiCl, 1 mM EDTA, 0.5% IGEPAL CA-63, and 0.5% sodium deoxycholate], and twice in TE buffer [10 mM tris-HCl (pH 8) and 1 mM EDTA]. Last, ChIP and input samples were subjected to ribonuclease digestion, proteinase K digestion, and reversal of cross-links at 65°C for 6 hours, before being purified with CleanNGS magnetic beads from CleanNA (GC Biotech B.V, Netherlands). The purified ChIP and input DNAs were sent to the Lausanne Genomic Technologies Facility for ChIP-seq library preparation using the NEBNext Ultra II DNA Library Prep Kit for Illumina and sequencing on two Illumina HiSeq lanes (150-bp paired-end).

### Centromere sequence identification

We first assessed whether specific genomic features (i.e., exons, introns, tandem repeats, DNA transposons, etc.; see Fig. 2B) were enriched in the ChIP data compared to the genomic background. This was done for the genome overall, as well as separately for the autosomes and the X chromosome. We trimmed ChIP and input reads using trimmomatic (version 0.39). Trimmed reads were then mapped to our reference genome using the BWA-MEM algorithm version 0.7.17 (-c 1000000000). Chimeric reads were removed using SA:Z tags, and PCR duplicates were eliminated with Picard (version 2.26.2). Mean coverage was computed for ChIP and input reads within nonoverlapping 10-kb windows across all scaffolds using BEDTools (version 2.30.0) and normalized by the number of mapped reads in each library. For each genome feature, we then summed the total length of all portions for which the mean ChIP coverage was at least 16× higher than the input coverage [i.e., log2(ChIP/input) ≥ 4]. The frequency of these enriched features was then compared to the frequencies of genome features in the assembly (for the whole genome, the autosomes or the X) using χ^2^ tests.

We found that tandem repeats were the major genomic feature enriched in the ChIP data (see main text and Fig. 2B). To characterize centromere sequences in *T. douglasi*, we therefore focused solely on tandem repeats. We used two different approaches, an assembly-based and an assembly-free approach. For the assembly-based approach, we defined enriched CenH3 windows (hereafter centromere windows) as 10-kb windows with a mean ChIP coverage at least four times higher than the mean input coverage [i.e., log2(ChIP/input) ≥ 2]. We filtered all genomic features from the centromere windows that were not tandem repeats or that were tandem repeats but with a local coverage log2(ChIP/input) ≤ 2. We then categorized these enriched tandem repeats into centromere repeat families in two steps. First, we generated a catalog of unique sequence motifs among the tandem repeats using a custom Perl script. This script identified minimal rotations (including reverse complements) of all repeat motifs found by TRF. In the second step, we investigated the sequence similarity of unique motifs by calculating a Levenshtein distance for each pairwise comparison. To facilitate comparisons, all motif sequences were adjusted to the size of the longest sequence by tandem duplications whereby we used a custom Python script to consider all rotations (i.e., all possible starting positions) of one of the two sequences and output the combination with the lowest Levenshtein distance. Pairwise comparisons of sequence motifs with at least 80% sequence similarity were further selected to build a network of sequence similarities from which distinct repeat families were defined (fig. S3A).

For the second approach to identify centromere repeat families, we used a k-mer–based analysis. We used the pipeline from (*72*) to construct k-mer databases for CenH3 ChIP-seq and input datasets with a k-mer length of 25 bp. A k-mer had to be found at least 100 times in the CenH3 and input datasets to be included in the k-mer database. We counted and normalized the abundance of each k-mer relative to the total base pairs. Centromere enrichment values were determined by calculating the ratio of normalized counts in the CenH3 dataset to those in the input dataset. Enriched k-mers were identified as those with a centromere enrichment score exceeding 25 median absolute deviations from the median (fig. S3B). CenH3-ChIP reads containing enriched k-mers were further assembled into de novo contigs using Spades version 3.15.3 (-careful) (*73*). We then annotated tandem repeats within these de novo contigs using TRF, following the same parameters as those used for annotating the genome (see above). Tandem repeat sequences identified in the de novo contigs were categorized into repeat families following the same methodology as in the assembly-based approach. In short, we generated a catalog of unique motif sequences, calculated a Levenshtein distance for each pairwise comparison, and built a network of sequence similarities (fig. S3C).

The assembly-based and assembly-free k-mer approaches identified a highly congruent set of centromere repeat families, with the families identified via the assembly-free approach representing a subset of those identified via the assembly-based approach (fig. S3 and tables S2 and S3). We therefore retained the centromere repeat families identified via the assembly-based approach for further analyses.

To examine the representation and organization of the centromere repeat families on each scaffold (including the shorter scaffolds not anchored to the 12 chromosomes), we used two different methods, with very similar results. For the first method, we summed, per scaffold, the TRF-inferred lengths of all repeat arrays for motifs belonging to a specific repeat family within centromere windows. For the second method, we conducted a BLASTN search of the motifs grouped into repeat families in the centromere windows. Blast hits with sequence identity and alignment coverage of the query below 80% were excluded. This alignment threshold was chosen to fit the alignment threshold applied by TRF to identify tandem copies (https://tandem.bu.edu/trf/desc). BLASTN hits were then assigned back to specific repeat families based on query identity (table S4) and visualized by extracting BLASTN hit coordinates within Geneious Prime (version 2023.1.1). The distribution and frequency of the centromere repeat families among scaffolds were then visualized using heatmaps (fig. S4, A and B).

### Design and selection of antibodies and probes

To design custom antibodies for *Timema* centromere proteins, we first used CenH3, CenpC, and Ndc80 protein sequences identified in (*38*) as initial queries to conduct tblastn searches against our *T. douglasi* gene annotations. For the CenH3 protein, three hits with E-values below 10-05 were identified, of which two were to the H3 and H3.3 histone units and were not considered further. The best hit corresponded to the CenH3 ortholog as revealed by Protein BLAST against the nonredundant NCBI database. For the CenpC and Ndc80 proteins, only a single hit with an E-value below 10-05 was identified. Last, we also conducted tblastn searches for the sequences of the three proteins against our *T. douglasi* gene annotations to identify possible paralogs, but only secondary hits with low percent sequence identity and coverage were recovered. We then used the three protein sequences to outsource to Covalab (Lyon, France) the design of three distinct peptide sequences for each protein (table S5). Follow-up tblastn searches against our *T. douglasi* gene annotations were performed to corroborate the absence of peptide cross-reactions with nontarget proteins. None of the peptides had cross-reactivity with other annotated genes for the CenH3 and Ndc80 proteins, while peptide 2 from the CenpC protein had a potential but unlikely cross-reactivity with the annotated, anonymous gene Tdi_034212-RA (i.e., 46% sequence identity). Covalab subsequently developed polyclonal rabbit antibodies for the three target genes using the designed peptides and purified them via a sepharose column.

We assessed the specificity of the CenH3 custom antibody using Western blot analyses. First, nuclear enriched proteins were extracted from frozen *Timema* testes. Testes were ground for 1 min at 25 Hz in liquid nitrogen using Cryomill (Retsch) and resuspended in 600 μl of TEB [PBS 1×, 0.5% Triton X-100 (v/v), and cOmplete Protease Inhibitor Cocktails (Sigma-Aldrich, #11697498001, one tab in 50 ml)]. After a short spin at 4°C to remove debris, the supernatant was transferred to a new tube and centrifuged for 10 min at 4°C at 2000 rpm. The supernatant was discarded and the pellet was resuspended in 40 μl of Laemmli buffer (1×) followed by 5-min incubation at 95°C. Final protein concentration was measured using tryptophan fluorescence according to (*74*).

Second, a Western blot was run from the nuclear enriched proteins and revealed a single band at the expected size for CenH3 (fig. S7). Nuclear-enriched proteins (1.5 μg) and prestained protein standard (Bio-Rad, #161-0377) were loaded into a 4 to 10% precast polyacrylamide gel (25 μl; Bio-Rad, #456-1093) and run for 30 min at 180 V in a Mini-trans-Blot Module following Bio-Rad recommendations. Proteins were then transferred to nitrocellulose membrane (Bio-Rad, #1620112) in wet conditions for 50 min at 4°C and 60 V according to Bio-Rad guidelines. A 5-min ponceau (Sigma-Aldrich, #P7170) staining was performed to check the quality of the transfer. A blocking step was performed using nonfat dry milk (NFDM) 3% in PBT (PBS 1× + 0.1% Tween-20) for 1 hour at RT. The membrane was then incubated overnight at 4°C with a solution of primary CenH3 antibody (diluted 1:3000 in NFDM 3%). Three washes of 10 min with PBT were performed before staining with anti-rabbit horseradish peroxidase secondary antibody (Jackson, #111-035-144, diluted 1:5000 in NFDM 3%) for 1 hour at RT. After three washes in PBT, Clarity Western ECL Substrate kit (Bio-Rad, #170-5060) was used for revelation, following instructions. Chemiluminescence acquisition of the membrane was made with a Fusion imaging system (by Vilber) with 20-s exposition.

To corroborate the centromere localizations of the CenH3-enriched motifs identified from the ChIP-seq data, we selected four of the most represented repeat families to design FISH probes that were custom-ordered from Microsynth (Balgach, Switzerland) and labeled using a 5′ modification with single fluorophore (table S5). In designing probes, we selected a representative motif within each of the four abundant families by trying to maximize its overall abundance (in base pairs) across centromere windows, the number of occurrences, and its connectivity within the network of CenH3-enriched motifs.

## References

[1] M. J. D. White, *Animal Cytology and Evolution 3rd ed*. (Cambridge, Eng.: University Press, 1973).

[2] D. K. Palmer, K. O’Day, H. L. Trong, H. Charbonneau, R. L. Margolis, Purification of the centromere-specific protein CENP-A and demonstration that it is a distinctive histone. Proc. Natl. Acad. Sci. U.S.A. 88, 3734–3738 (1991). 2023923 10.1073/pnas.88.9.3734 PMC51527

[3] K. Yoda, S. Ando, S. Morishita, K. Houmura, K. Hashimoto, K. Takeyasu, T. Okazaki, Human centromere protein A (CENP-A) can replace histone H3 in nucleosome reconstitution. Proc. Natl. Acad. Sci. U.S.A. 97, 7266–7271 (2000). 10840064 10.1073/pnas.130189697 PMC16534

[4] D. W. Cleveland, Y. Mao, K. F. Sullivan, Centromeres and kinetochores: From epigenetics to mitotic checkpoint signaling. Cell 112, 407–421 (2003). 12600307 10.1016/s0092-8674(03)00115-6

[5] K. L. McKinley, I. M. Cheeseman, The molecular basis for centromere identity and function. Nat. Rev. Mol. Cell Biol. 17, 16–29 (2016). 26601620 10.1038/nrm.2015.5 PMC8603311

[6] A. Musacchio, A. Desai, A molecular view of kinetochore assembly and function. Biology 6, 5 (2017). 28125021 10.3390/biology6010005 PMC5371998

[7] P. B. Talbert, S. Henikoff, What makes a centromere? Exp. Cell Res. 389, 111895 (2020). 32035948 10.1016/j.yexcr.2020.111895

[8] V. Schubert, P. Neumann, A. Marques, S. Heckmann, J. Macas, A. Pedrosa-Harand, I. Schubert, T.-S. Jang, A. Houben, Super-resolution microscopy reveals diversity of plant centromere architecture. Int. J. Mol. Sci. 21, 3488 (2020). 32429054 10.3390/ijms21103488 PMC7278974

[9] A. P. Senaratne, N. Cortes-Silva, I. A. Drinnenberg, Evolution of holocentric chromosomes: Drivers, diversity, and deterrents. Semin. Cell Dev. Biol. 127, 90–99 (2022). 35031207 10.1016/j.semcdb.2022.01.003

[10] Y.-T. Kuo, A. S. Câmara, V. Schubert, P. Neumann, J. Macas, M. Melzer, J. Chen, J. Fuchs, S. Abel, E. Klocke, B. Huettel, A. Himmelbach, D. Demidov, F. Dunemann, M. Mascher, T. Ishii, A. Marques, A. Houben, Holocentromeres can consist of merely a few megabase-sized satellite arrays. Nat. Commun. 14, 3502 (2023). 37311740 10.1038/s41467-023-38922-7 PMC10264360

[11] F. A. Steiner, S. Henikoff, Holocentromeres are dispersed point centromeres localized at transcription factor hotspots. eLife 3, e02025 (2014). 24714495 10.7554/eLife.02025 PMC3975580

[12] R. Gassmann, A. Rechtsteiner, K. W. Yuen, A. Muroyama, T. Egelhofer, L. Gaydos, F. Barron, P. Maddox, A. Essex, J. Monen, S. Ercan, J. D. Lieb, K. Oegema, S. Strome, A. Desai, An inverse relationship to germline transcription defines centromeric chromatin in *C. elegans*. Nature 484, 534–537 (2012). 22495302 10.1038/nature10973 PMC3538161

[13] A. P. Senaratne, H. Muller, K. A. Fryer, M. Kawamoto, S. Katsuma, I. A. Drinnenberg, Formation of the CenH3-deficient holocentromere in lepidoptera avoids active chromatin. Curr. Biol. 31, 173–181.e7 (2021). 33125865 10.1016/j.cub.2020.09.078

[14] Y. Kang, J. Wang, A. Neff, S. Kratzer, H. Kimura, R. E. Davis, Differential chromosomal localization of centromeric histone CENP-A contributes to nematode programmed DNA elimination. Cell Rep. 16, 2308–2316 (2016). 27545882 10.1016/j.celrep.2016.07.079 PMC5007152

[15] H. S. Malik, S. Henikoff, Major evolutionary transitions in centromere complexity. Cell 138, 1067–1082 (2009). 19766562 10.1016/j.cell.2009.08.036

[16] T. Schwander, L. Henry, B. J. Crespi, Molecular evidence for ancient asexuality in Timema stick insects. Curr. Biol. 21, 1129–1134 (2011). 21683598 10.1016/j.cub.2011.05.026

[17] T. Schwander, B. J. Crespi, Multiple direct transitions from sexual reproduction to apomictic parthenogenesis in Timema stick insects. Evolution 63, 84–103 (2009). 18803687 10.1111/j.1558-5646.2008.00524.x

[18] W. Ma, V. Schubert, M. M. Martis, G. Hause, Z. Liu, Y. Shen, U. Conrad, W. Shi, U. Scholz, S. Taudien, Z. Cheng, A. Houben, A. Houben, The distribution of α-kleisin during meiosis in the holocentromeric plant *Luzula elegans*. Chromosome Res. 24, 393–405 (2016). 27294972 10.1007/s10577-016-9529-5

[19] B. J. Buchwitz, K. Ahmad, L. L. Moore, M. B. Roth, S. Henikoff, Cell division: A histone-H3-like protein in *C. elegans*. Nature 401, 547–548 (1999). 10524621 10.1038/44062

[20] A. M. Valdeolmillos, A. Viera, J. Page, I. Prieto, J. L. Santos, M. T. Parra, M. M. S. Heck, C. Martínez-A, J. L. Barbero, J. A. Suja, J. S. Rufas, Sequential loading of cohesin subunits during the first meiotic prophase of grasshoppers. PLOS Genet. 3, 204–215 (2007). 10.1371/journal.pgen.0030028 PMC1802827 17319746

[21] C. Grey, B. de Massy, Chromosome organization in early meiotic prophase. Front. Cell Dev. Biol. 9, 688878 (2021). 34150782 10.3389/fcell.2021.688878 PMC8209517

[22] C. Hockens, H. Lorenzi, T. T. Wang, E. P. Lei, L. F. Rosin, Chromosome segregation during spermatogenesis occurs through a unique center-kinetic mechanism in holocentric moth species. PLOS Genet. 20, e1011329 (2024). 38913752 10.1371/journal.pgen.1011329 PMC11226059

[23] Y. Xiang, D. Tsuchiya, Z. Yu, X. Zhao, S. McKinney, J. Unruh, B. Slaughter, C. M. Lake, R. S. Hawley, Multiple reorganizations of the lateral elements of the synaptonemal complex facilitate homolog segregation in *Bombyx mori* oocytes. Curr. Biol. 34, 352–360.e4 (2024). 38176417 10.1016/j.cub.2023.12.018

[24] R. Frydrychová, P. Grossmann, P. Trubac, M. Vitková, F. E. Marec, Phylogenetic distribution of TTAGG telomeric repeats in insects. Genome 47, 163–178 (2004). 15060613 10.1139/g03-100

[25] T. Liehr, O. Buleu, T. Karamysheva, A. Bugrov, N. Rubtsov, New insights into Phasmatodea chromosomes. Genes 8, 11 (2017). 10.3390/genes8110327 PMC5704240 29149047

[26] S. Henikoff, K. Ahmad, H. S. Malik, The centromere paradox: Stable inheritance with rapidly evolving DNA. Science 293, 1098–1102 (2001). 11498581 10.1126/science.1062939

[27] E. M. Hildebrand, J. Dekker, Mechanisms and functions of chromosome compartmentalization. Trends Biochem. Sci. 45, 385–396 (2020). 32311333 10.1016/j.tibs.2020.01.002 PMC7275117

[28] H. Muller, J. Gil Jr., I. A. Drinnenberg, The impact of centromeres on spatial genome architecture. Trends Genet. 35, 565–578 (2019). 31200946 10.1016/j.tig.2019.05.003

[29] B. G. Mellone, D. Fachinetti, Diverse mechanisms of centromere specification. Curr. Biol. 31, R1491–R1504 (2021). 34813757 10.1016/j.cub.2021.09.083 PMC8820161

[30] R. F. Prosée, J. M. Wenda, F. A. Steiner, Adaptations for centromere function in meiosis. Essays Biochem. 64, 193–203 (2020). 32406496 10.1042/EBC20190076 PMC7475650

[31] L. Schermelleh, A. Ferrand, T. Huser, C. Eggeling, M. Sauer, O. Biehlmaier, G. P. C. Drummen, Super-resolution microscopy demystified. Nat. Cell Biol. 21, 72–84 (2019). 30602772 10.1038/s41556-018-0251-8

[32] G. Fabig, T. Müller-Reichert, L. V. Paliulis, Back to the roots: Segregation of univalent sex chromosomes in meiosis. Chromosoma 125, 277–286 (2016). 26511278 10.1007/s00412-015-0550-9

[33] R. B. Nicklas, J. C. Waters, E. D. Salmon, S. C. Ward, Checkpoint signals in grasshopper meiosis are sensitive to microtubule attachment, but tension is still essential. J. Cell Sci. 114, 4173–4183 (2001). 11739650 10.1242/jcs.114.23.4173

[34] A. A. Van Hooser, I. I. Ouspenski, H. C. Gregson, D. A. Starr, T. J. Yen, M. L. Goldberg, K. Yokomori, W. C. Earnshaw, K. F. Sullivan, B. R. Brinkley, Specification of kinetochore-forming chromatin by the histone H3 variant CENP-A. J. Cell Sci. 114, 3529–3542 (2001). 11682612 10.1242/jcs.114.19.3529

[35] P. Heun, S. Erhardt, M. D. Blower, S. Weiss, A. D. Skora, G. H. Karpen, Mislocalization of the *Drosophila* centromere-specific histone CID promotes formation of functional ectopic kinetochores. Dev. Cell 10, 303–315 (2006). 16516834 10.1016/j.devcel.2006.01.014 PMC3192491

[36] S. Heckmann, M. Jankowska, V. Schubert, K. Kumke, W. Ma, A. Houben, Alternative meiotic chromatid segregation in the holocentric plant *Luzula elegans*. Nat. Commun. 5, 4979 (2014). 25296379 10.1038/ncomms5979 PMC4214429

[37] N. Cortes-Silva, J. Ulmer, T. Kiuchi, E. Hsieh, G. Cornilleau, I. Ladid, F. Dingli, D. Loew, S. Katsuma, I. A. Drinnenberg, CenH3-independent kinetochore assembly in Lepidoptera requires CCAN, including CENP-T. Curr. Biol. 30, 561–572.e10 (2020). 32032508 10.1016/j.cub.2019.12.014

[38] I. A. Drinnenberg, D. deYoung, S. Henikoff, H. S. Malik, Recurrent loss of CenH3 is associated with independent transitions to holocentricity in insects. eLife 3, e03676 (2014). 25247700 10.7554/eLife.03676 PMC4359364

[39] P. Neumann, L. Oliveira, T. S. Jang, P. Novák, A. Koblizková, V. Schubert, A. Houben, J. Macas, Disruption of the standard kinetochore in holocentric Cuscuta species. Proc. Natl. Acad. Sci. U.S.A. 120, e2300877120 (2023). 37192159 10.1073/pnas.2300877120 PMC10214151

[40] C. Courret, L. Hemmer, X. Wei, P. D. Patel, B. Santinello, X. Geng, C.-H. Chang, B. Mellone, A. M. Larracuente, Rapid turnover of centromeric DNA reveals signatures of genetic conflict in Drosophila. bioRxiv 554357 [Preprint], (2023) https://www.biorxiv.org/content/10.1101/2023.08.22.554357v1.

[41] L. A. Robledillo, P. Neumann, A. Koblízková, P. Novák, I. Vrbová, J. Macas, Extraordinary sequence diversity and promiscuity of centromeric satellites in the legume tribe. Mol. Biol. Evol. 37, 2341–2356 (2020). 32259249 10.1093/molbev/msaa090 PMC7403623

[42] Z. Gong, Y. Wu, A. Koblízková, G. A. Torres, K. Wang, M. Iovene, P. Neumann, W. Zhang, P. Novák, C. R. Buell, J. Macas, J. Jiang, Repeatless and repeat-based centromeres in potato: Implications for centromere evolution. Plant Cell 24, 3559–3574 (2012). 22968715 10.1105/tpc.112.100511 PMC3480287

[43] W.-H. Shang, T. Hori, A. Toyoda, J. Kato, K. Popendorf, Y. Sakakibara, A. Fujiyama, T. Fukagawa, Chickens possess centromeres with both extended tandem repeats and short non-tandem-repetitive sequences. Genome Res. 20, 1219–1228 (2010). 20534883 10.1101/gr.106245.110 PMC2928500

[44] M. Kolmogorov, J. Yuan, Y. Lin, P. A. Pevzner, Assembly of long, error-prone reads using repeat graphs. Nat. Biotechnol. 37, 540–546 (2019). 30936562 10.1038/s41587-019-0072-8

[45] H. Li, Minimap2: Pairwise alignment for nucleotide sequences. Bioinformatics 34, 3094–3100 (2018). 29750242 10.1093/bioinformatics/bty191 PMC6137996

[46] R. Vaser, I. Sovic, N. Nagarajan, M. Sikic, Fast and accurate de novo genome assembly from long uncorrected reads. Genome Res. 27, 737–746 (2017). 28100585 10.1101/gr.214270.116 PMC5411768

[47] H. Li, Aligning sequence reads, clone sequences and assembly contigs with BWA-MEM. arXiv:1303.3997 (2013); https://arxiv.org/abs/1303.3997.

[48] B. J. Walker, T. Abeel, T. Shea, M. Priest, A. Abouelliel, S. Sakthikumar, C. A. Cuomo, Q. Zeng, J. Wortman, S. K. Young, A. M. Earl, Pilon: An integrated tool for comprehensive microbial variant detection and genome assembly improvement. PLOS ONE 9, e112963 (2014). 25409509 10.1371/journal.pone.0112963 PMC4237348

[49] D.R. Laetsch, M. L. Blaxter, BlobTools: Interrogation of genome assemblies [version 1; peer review: 2 approved with reservations]. F1000Research. 2017.

[50] M. J. Roach, S. A. Schmidt, A. R. Borneman, Purge haplotigs: Allelic contig reassignment for third-gen diploid genome assemblies. BMC Bioinformatics 19, 460 (2018). 30497373 10.1186/s12859-018-2485-7 PMC6267036

[51] N. C. Durand, M. S. Shamim, I. Machol, S. S. P. Rao, M. H. Huntley, E. S. Lander, E. L. Aiden, Juicer provides a one-click system for analyzing loop-resolution Hi-C experiments. Cell Syst. 3, 95–98 (2016). 27467249 10.1016/j.cels.2016.07.002 PMC5846465

[52] O. Dudchenko, S. S. Batra, A. D. Omer, S. K. Nyquist, M. Hoeger, N. C. Durand, M. S. Shamim, I. Machol, E. S. Lander, A. P. Aiden, E. L. Aiden, De novo assembly of the Aedes aegypti genome using Hi-C yields chromosome-length scaffolds. Science 356, 92–95 (2017). 28336562 10.1126/science.aal3327 PMC5635820

[53] M. Seppey, M. Manni, E. M. Zdobnov, BUSCO: Assessing genome assembly and annotation completeness. Methods Mol. Biol. 1962, 227–245 (2019). 31020564 10.1007/978-1-4939-9173-0_14

[54] K. S. Jaron, D. J. Parker, Y. Anselmetti, P. T. Van, J. Bast, Z. Dumas, E. Figuet, C. M. François, K. Hayward, V. Rossier, P. Simion, M. Robinson-Rechavi, N. Galtier, T. Schwander, Convergent consequences of parthenogenesis on stick insect genomes. Sci. Adv. 8, eabg3842 (2022). 35196080 10.1126/sciadv.abg3842 PMC8865771

[55] C. Larose, G. Lavanchy, S. Freitas, D. J. Parker, T. Schwander, Facultative parthenogenesis: A transient state in transitions between sex and obligate asexuality in stick insects? Peer Commun. J. 3, e60 (2023).

[56] T. Bruna, K. J. Hoff, A. Lomsadze, M. Stanke, M. Borodovsky, BRAKER2: Automatic eukaryotic genome annotation with GeneMark-EP+ and AUGUSTUS supported by a protein database. NAR Genom. Bioinform. 3, lqaa108 (2021). 33575650 10.1093/nargab/lqaa108 PMC7787252

[57] E. V. Kriventseva, D. Kuznetsov, F. Tegenfeldt, M. Manni, R. Dias, F. A. Simão, E. M. Zdobnov, OrthoDB v10: Sampling the diversity of animal, plant, fungal, protist, bacterial and viral genomes for evolutionary and functional annotations of orthologs. Nucleic Acids Res. 47, D807–D811 (2019). 30395283 10.1093/nar/gky1053 PMC6323947

[58] A. M. Bolger, M. Lohse, B. Usadel, Trimmomatic: A flexible trimmer for Illumina sequence data. Bioinformatics 30, 2114–2120 (2014). 24695404 10.1093/bioinformatics/btu170 PMC4103590

[59] A. Dobin, C. A. Davis, F. Schlesinger, J. Drenkow, C. Zaleski, S. Jha, P. Batut, M. Chaisson, T. R. Gingeras, STAR: Ultrafast universal RNA-seq aligner. Bioinformatics 29, 15–21 (2013). 23104886 10.1093/bioinformatics/bts635 PMC3530905

[60] M. Stanke, M. Diekhans, R. Baertsch, D. Haussler, Using native and syntenically mapped cDNA alignments to improve de novo gene finding. Bioinformatics 24, 637–644 (2008). 18218656 10.1093/bioinformatics/btn013

[61] T. Bruna, A. Lomsadze, M. Borodovsky, GeneMark-EP+: Eukaryotic gene prediction with self-training in the space of genes and proteins. NAR Genom. Bioinform. 2, lqaa026 (2020). 32440658 10.1093/nargab/lqaa026 PMC7222226

[62] J. Keilwagen, F. Hartung, J. Grau, GeMoMa: Homology-based gene prediction utilizing intron position conservation and RNA-seq data. Methods Mol. Biol. 1962, 161–177 (2019). 31020559 10.1007/978-1-4939-9173-0_9

[63] L. Gabriel, K. J. Hoff, T. Bruna, M. Borodovsky, M. Stanke, TSEBRA: Transcript selector for BRAKER. BMC Bioinformatics 22, 566 (2021). 34823473 10.1186/s12859-021-04482-0 PMC8620231

[64] E. P. Nawrocki, S. R. Eddy, Infernal 1.1: 100-fold faster RNA homology searches. Bioinformatics 29, 2933–2935 (2013). 24008419 10.1093/bioinformatics/btt509 PMC3810854

[65] J. M. Flynn, R. Hubley, C. Goubert, J. Rosen, A. G. Clark, C. Feschotte, A. F. Smit, RepeatModeler2 for automated genomic discovery of transposable element families. Proc. Natl. Acad. Sci. U.S.A. 117, 9451–9457 (2020). 32300014 10.1073/pnas.1921046117 PMC7196820

[66] M. Steinegger, J. Söding, MMseqs2 enables sensitive protein sequence searching for the analysis of massive data sets. Nat. Biotechnol. 35, 1026–1028 (2017). 29035372 10.1038/nbt.3988

[67] A. Smit, Hubley, R & Green, P. RepeatMasker Open-4.0. 2013–2015; http://www.repeatmasker.org.

[68] G. Benson, Tandem repeats finder: A program to analyze DNA sequences. Nucleic Acids Res. 27, 573–580 (1999). 9862982 10.1093/nar/27.2.573 PMC148217

[69] J. Schindelin, I. Arganda-Carreras, E. Frise, V. Kaynig, M. Longair, T. Pietzsch, S. Preibisch, C. Rueden, S. Saalfeld, B. Schmid, J.-Y. Tinevez, D. J. White, V. Hartenstein, K. Eliceiri, P. Tomancak, A. Cardona, Fiji: An open-source platform for biological-image analysis. Nat. Methods 9, 676–682 (2012). 22743772 10.1038/nmeth.2019 PMC3855844

[70] I. Kubalova, A. Nemeckova, K. Weisshart, E. Hribova, V. Schubert, Comparing super-resolution microscopy techniques to analyze chromosomes. Int. J. Mol. Sci. 22, 1903 (2021). 33672992 10.3390/ijms22041903 PMC7917581

[71] K. Weisshart, J. Fuchs, V. Schubert, Structured illumination microscopy (SIM) and photoactivated localization microscopy (PALM) to analyze the abundance and distribution of RNA polymerase II molecules in flow-sorted Arabidopsis nuclei. Bio Protocol 6, e1725 (2016).

[72] O. K. Smith, C. Limouse, K. A. Fryer, N. A. Teran, K. Sundararajan, R. Heald, A. F. Straight, Identification and characterization of centromeric sequences in *Xenopus laevis*. Genome Res. 31, 958–967 (2021). 33875480 10.1101/gr.267781.120 PMC8168581

[73] S. Nurk, A. Bankevich, D. Antipov, A. A. Gurevich, A. Korobeynikov, A. Lapidus, A. D. Prjibelski, A. Pyshkin, A. Sirotkin, Y. Sirotkin, R. Stepanauskas, S. R. Clingenpeel, T. Woyke, J. S. M. Lean, R. Lasken, G. Tesler, M. A. Alekseyev, P. A. Pevzner, Assembling single-cell genomes and mini-metagenomes from chimeric MDA products. J. Comput. Biol. 20, 714–737 (2013). 24093227 10.1089/cmb.2013.0084 PMC3791033

[74] J. R. Wisniewski, F. Z. Gaugaz, Fast and sensitive total protein and peptide assays for proteomic analysis. Anal. Chem. 87, 4110–4116 (2015). 25837572 10.1021/ac504689z

