# Supplementary material for: Functional monocentricity with holocentric characteristics and chromosome-specific centromeres in a stick insect
Source: Sci Adv. 2025 Jan 1;11(1):eads6459. doi: 10.1126/sciadv.ads6459 (PMC11691646; doi:10.1126/sciadv.ads6459)
Supplement: Supplementary file 1 — Figs. S1 to S7 Tables S1 and S5 Legends for tables S2 to S4 [file sciadv.ads6459_sm.pdf]

Science Advances
AAAS

Supplementary Materials for

Functional monocentricity with holocentric characteristics and chromosome-specific centromeres in a stick insect

William Toubiana *et al.*

Corresponding author: William Toubiana, toubianawilliam@gmail.com;
Tanja Schwander, tanja.schwander@unil.ch

Sci. Adv. **11**, eads6459 (2025)
DOI: 10.1126/sciadv.ads6459

The PDF file includes:

Figs. S1 to S7
Tables S1 and S5
Legends for tables S2 to S4

Other Supplementary Material for this manuscript includes the following:

Tables S2 to S4

Supplemental Figures

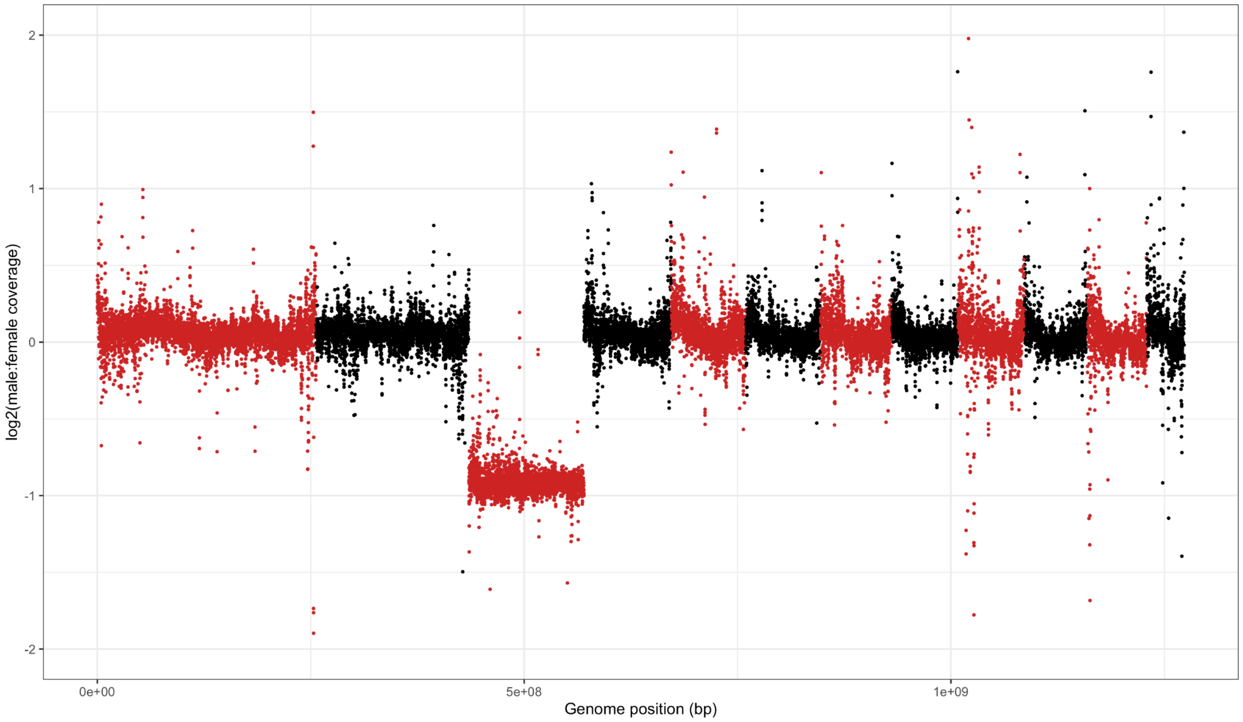

figure S1. *T. douglasi* X chromosome identification. The plot shows the $\log_2$ ratio of male to female coverage of 100 kb sliding windows across the genome. Alternated colors designate different chromosomes, with chromosome 3 showing a much lower overall male to female coverage ratio.

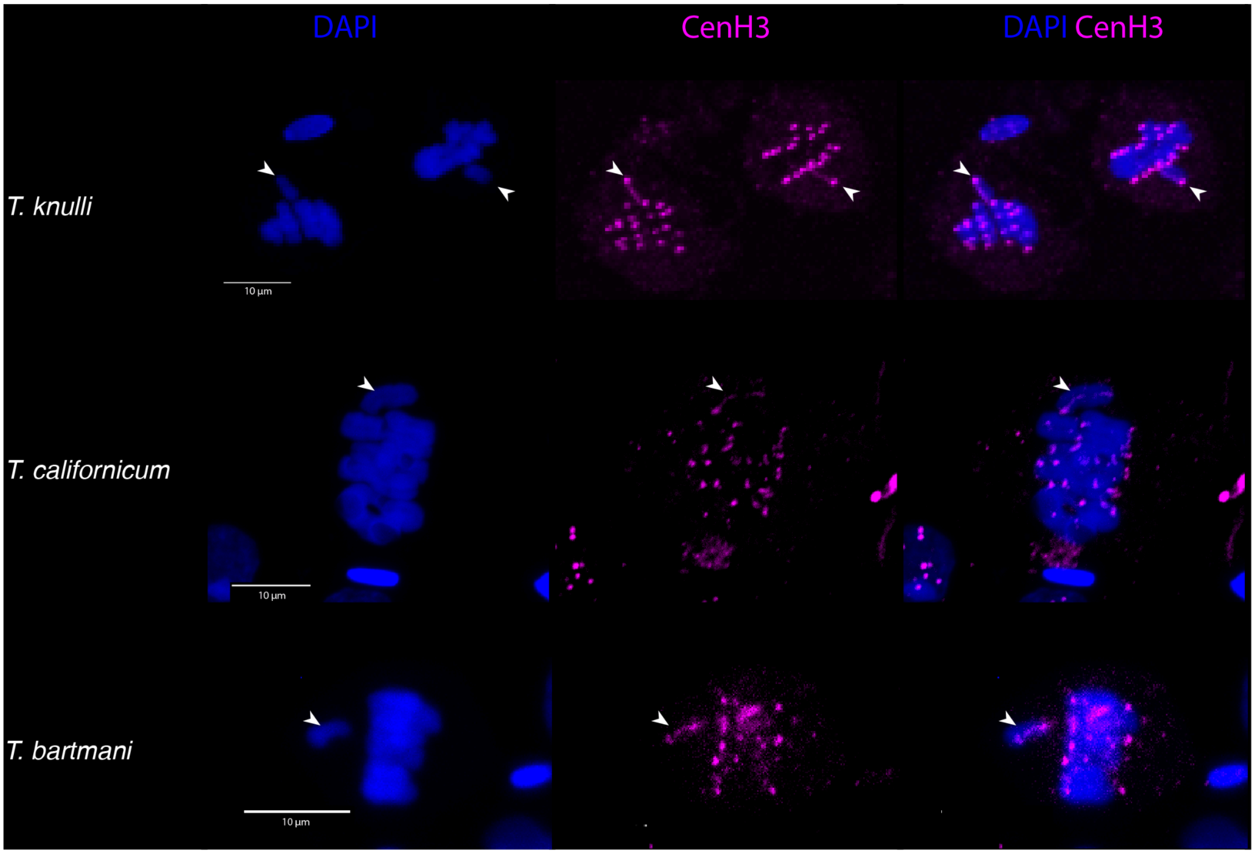

figure S2. Monocentric and longitudinal CenH3 binding along autosomes and the X chromosome, respectively, for meiotic cells of males from three different *Timema* species.

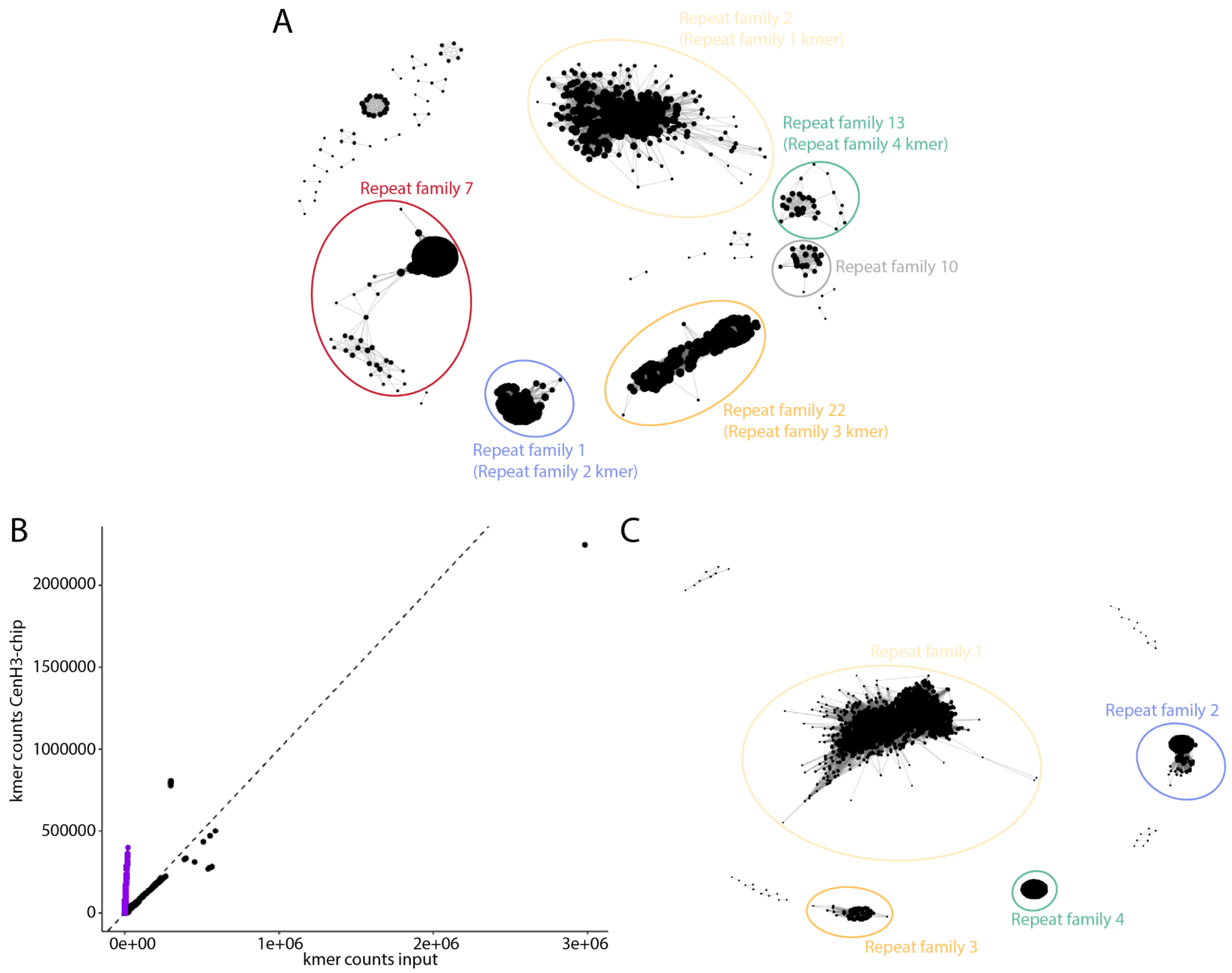

figure S3. Identification of centromere sequences. A) Network of sequence similarities among tandem repeat motifs identified in the genome assembly. In the two networks, each node represents a unique CenH3-enriched motif sequence and edges connect motifs with at least 80% sequence similarities. B) Scatterplot of 25-bp k-mer normalized counts found in input and CenH3 ChIP-seq libraries. Enriched k-mers are highlighted in purple and were identified as those with a centromere enrichment score exceeding 25 absolute deviations from the median. C) Network of sequence similarities among tandem repeat motifs identified in *de novo* contigs (k-mer based approach).

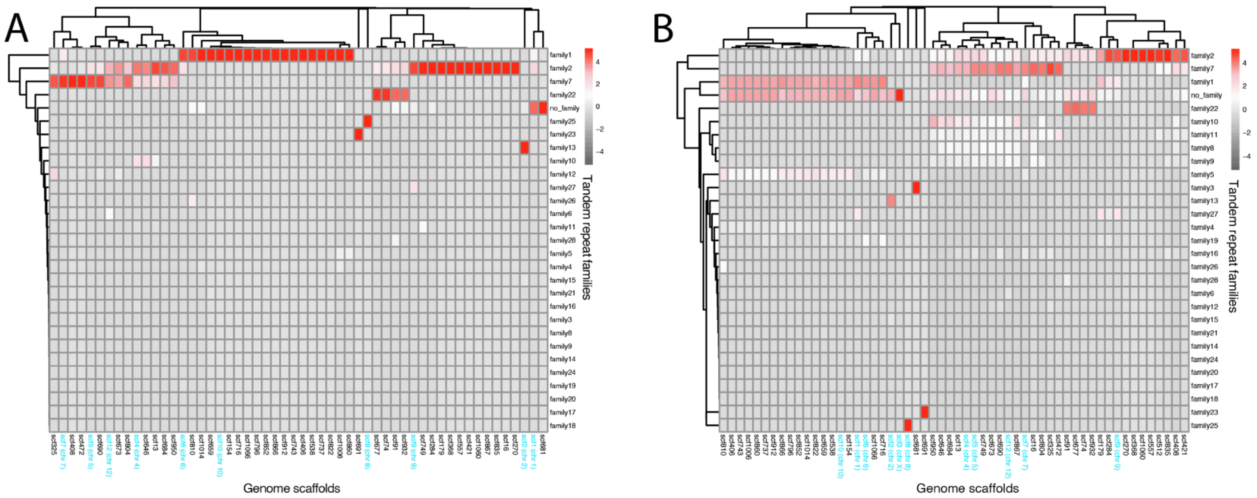

figure S4. Centromeres of different chromosomes consist of distinct tandem repeat families. The heatmaps are based on the subset of scaffolds comprising centromere tandem repeat families in at least one centromere window, with hierarchical clustering based on (A) total array length inferred by summing Tandem Repeat Finder array lengths per repeat family and (B) total array length inferred by summing the lengths of sequence motif blast hits with 80% sequence similarity and 80% query coverage. Chromosome scaffolds are highlighted in cyan.

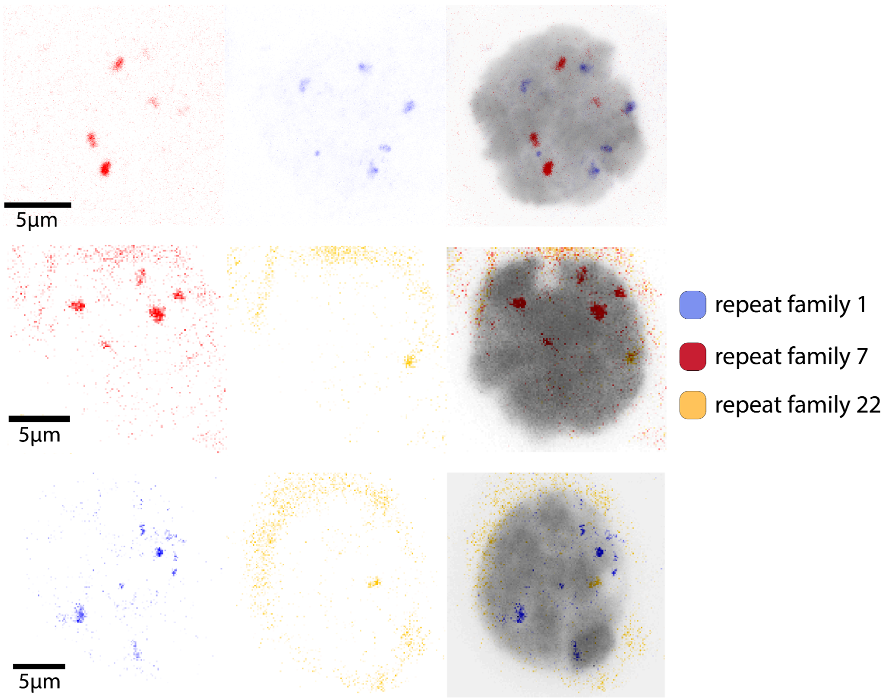

figure S5. Fluorescent In Situ Hybridization of DNA probes labeling specific centromere repeat families. DNA is labeled with DAPI in grey.

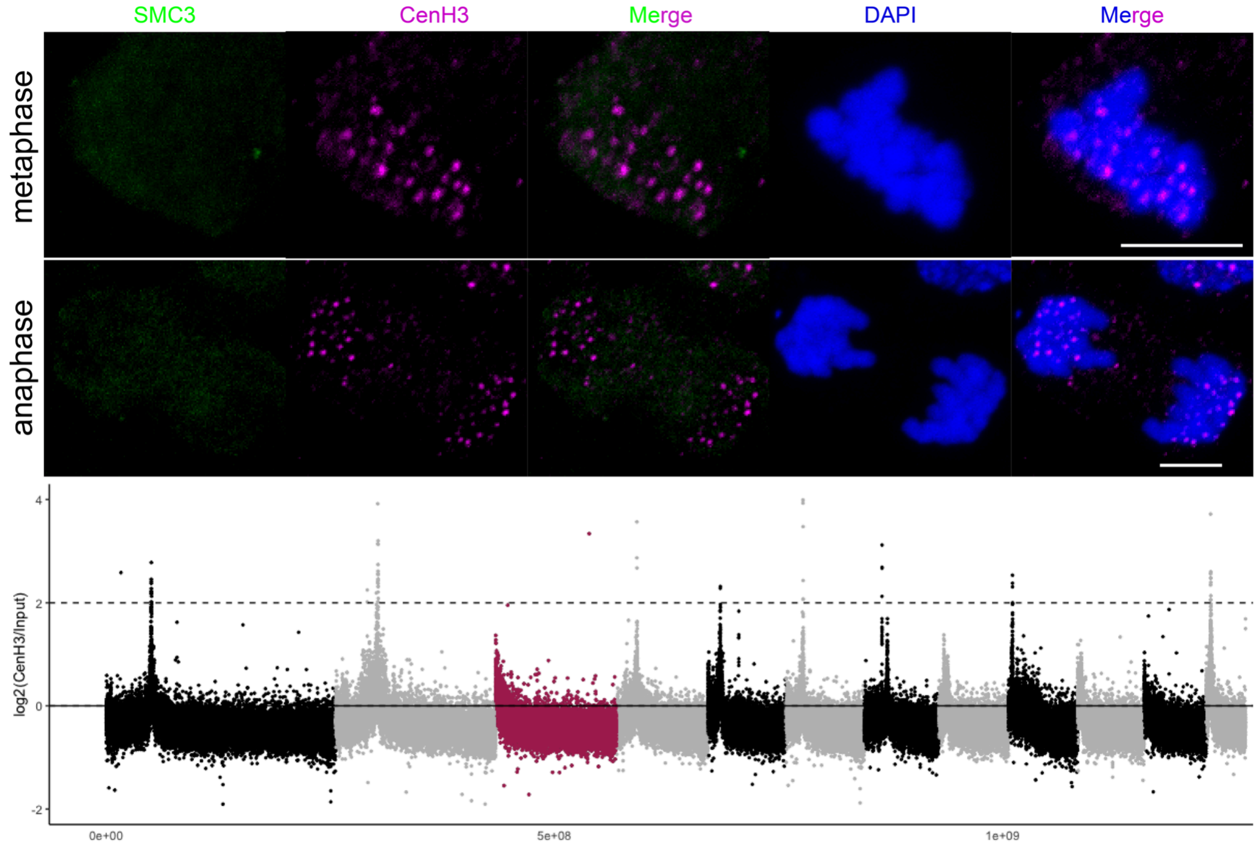

figure S6. CenH3 binding in mitotic and somatic cells. Upper panel: Monocentric CenH3 binding on all chromosomes for mitotic cells (i.e., metaphase and anaphase) of males from *T. californicum*. Scale bars: 5 µm. Bottom panel: CenH3-direct ChIP-signal on somatic tissues from *T. douglasi* females.

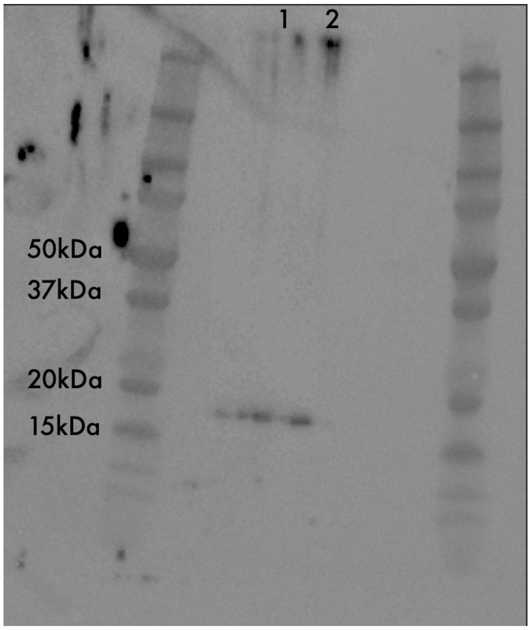

figure S7. Immunoblotting using the custom CenH3 antibody. Immunoblot analysis of protein extracts from *Timema* testes, using the custom polyclonal antibody against CenH3. Molecular weight markers are indicated by numbers in kilodaltons (*left*) and their position by lines. The CenH3 polyclonal antibody is expected to recognise a band around 18 kDa in *Timema*.

Supplemental tables

table S1. *T. douglasi* genome assembly statistics. The size of the *T. douglasi* genome assembly is approximately 1.29 Gb, fitting well with estimates from previous short-read assemblies (55), and high BUSCO scores reveal that it is largely complete. The number of large scaffolds assembled (n=12) matches the number of chromosomes inferred from karyotypes (11 autosomes and 1 X chromosome; (17)) and comprises 98.4% of the total assembly. The annotation of the total assembly includes 39688 genes, 32.2% of TEs and 22.6% of TRs.

Species (accession number)	Total assembly size (all scaffolds)	Size of the 12 large scaffolds	Total number of scaffolds	BUSCO v5, n:1367
T. douglasi	1.29 Gbp	1.27 Gbp	1136	C:99.0%[S:98.2%,

(PRJNA1123914)				D:0.8%],F:0.7%,M :0.3%<br>4 missing.

table S2. Tandem repeat motifs assembly-based method.

table S3. Tandem repeat motifs reference-free method.

table S4. Blast hits of tandem repeat motifs against centromere windows.

table S5. Antibodies and probes used. A combination of custom and commercially available antibodies as well as DNA probes was employed for the immunostaining and FISH assays. The CenH3-custom antibody was further used in the ChIP-seq and ImmunoFISH experiments.

List of primary custom antibodies			
**Short Name**	**Finale Concentration**	**Peptide sequence**	**Tdi gene annotation reference**
CenH3	1/100	VRRKSSAKKRSIRISGPREET-C-coOH	Tdi_018724-RA
		C-ETSARSNKTQNDSSKPSTSH-coNH2	
		C-SKPSTSHHKSKNKSTRWSG-coNH2	
CenPC	1/100	C-ESSVREVTKSSSGGS-coNH2	Tdi_024923-RA
		C-KVHNKTKQTSKGRNKT-coNH2	
		C-TYTKHGAEELSGSGE-coNH2	
NDC80	1/100	C-FSSNKGSQKNKNTYAT-coNH2	Tdi_023905-RA
		C-DKVGFSEAEEQKYLE-coNH2	
		C-KEESAQAEYKQEREK-coNH2	
List of primary commercial antibodies			
**Short Name**	**Reference**	**Company**	**Finale Concentration**
SMC3	ab201542	AbCam	1/100
α_Tubulin	#F2168	Sigma-Aldrich	1/150
List of secondary antibodies			
**Short Name**	**Reference**	**Company**	**Finale Concentration**
anti-Rabbit-Alexa 594	711-585-152	Jackson	1/150
List of probes			
**Short Name**	**5' modification**	**Probe sequence**	
Repeat family 1	Alexa fluorophore 488	GAAGATTATTGAAATCAAGTATGTCCGCTTTGTTGATTATTTTCCG	
Repeat family 7	Fluorophore Cy5	CTAAATAATCAAAATCGGCTATGGATGCTCGGTTGACAAGTTTTATC	

Repeat family 13	Alexa fluorophore 594	AGCTGAAGTGTGCAAGTATTTGTGGGAGGATACCATACCCGGCGTCTAGT	
Repeat family 22	Alexa fluorophore 594	TAGTTAGGCTAGATCAGCCAGTCAGGTCGTATCCCCGTATCTTATAGAGT	
